# Segregation of Fluorescent Membrane Lipids into Distinct Micrometric Domains: Evidence for Phase Compartmentation of Natural Lipids?

**DOI:** 10.1371/journal.pone.0017021

**Published:** 2011-02-28

**Authors:** Ludovic D′auria, Patrick Van Der Smissen, Frédéric Bruyneel, Pierre J. Courtoy, Donatienne Tyteca

**Affiliations:** 1 CELL Unit, de Duve Institute and Université catholique de Louvain, Brussels, Belgium; 2 CHOM Unit, Université catholique de Louvain, Louvain-la-Neuve, Belgium; Cornell University, United States of America

## Abstract

**Background:**

We recently reported that sphingomyelin (SM) analogs substituted on the alkyl chain by various fluorophores (e.g. BODIPY) readily inserted at trace levels into the plasma membrane of living erythrocytes or CHO cells and spontaneously concentrated into micrometric domains. Despite sharing the same fluorescent ceramide backbone, BODIPY-SM domains segregated from similar domains labelled by BODIPY-D-*e*-lactosylceramide (D-*e*-LacCer) and depended on endogenous SM.

**Methodology/Principal Findings:**

We show here that BODIPY-SM further differed from BODIPY-D-*e*-LacCer or -glucosylceramide (GlcCer) domains in temperature dependence, propensity to excimer formation, association with a glycosylphosphatidylinositol (GPI)-anchored fluorescent protein reporter, and lateral diffusion by FRAP, thus demonstrating different lipid phases and boundaries. Whereas BODIPY-D-*e*-LacCer behaved like BODIPY-GlcCer, its artificial stereoisomer, BODIPY-L-*t*-LacCer, behaved like BODIPY- and NBD-phosphatidylcholine (PC). Surprisingly, these two PC analogs also formed micrometric patches yet preferably at low temperature, did not show excimer, never associated with the GPI reporter and showed major restriction to lateral diffusion when photobleached in large fields. This functional comparison supported a three-phase micrometric compartmentation, of decreasing order: BODIPY-GSLs > -SM > -PC (or artificial L-*t*-LacCer). Co-existence of three segregated compartments was further supported by double labelling experiments and was confirmed by additive occupancy, up to ∼70% cell surface coverage. Specific alterations of BODIPY-analogs domains by manipulation of corresponding endogenous sphingolipids suggested that distinct fluorescent lipid partition might reflect differential intrinsic propensity of endogenous membrane lipids to form large assemblies.

**Conclusions/Significance:**

We conclude that fluorescent membrane lipids spontaneously concentrate into distinct micrometric assemblies. We hypothesize that these might reflect preexisting compartmentation of endogenous PM lipids into non-overlapping domains of differential order: GSLs > SM > PC, resulting into differential self-adhesion of the two former, with exclusion of the latter.

## Introduction

Biological membranes are organized as lipid bilayers, initially represented as homogenous solvent for membrane proteins [1]. The major structural lipids in mammalian cell membranes are glycerophospholipids, sphingolipids (SLs) and cholesterol. The most abundant glycerophospholipid is phosphatidylcholine (PC), whose frequently unsaturated fatty acid induces bending within the lipid phase, causing disordered packaging, referred to as “liquid-disordered state” (L_d_). Although sharing overall similarity in three-dimensional structure with glycerophospholipids, SLs are built up from the very different ceramide backbone, bearing two long parallel alkyl chains, favouring ordered assembly, referred to as “liquid-ordered state” (L_o_). SLs include the zwitterionic sphingomyelin (SM), bearing the same phosphocholine headgroup as PC, and glycosphingolipids (GSLs), an heterogeneous family comprising mono- (e.g. glucosylceramide; GlcCer), di- (e.g, D-*e*-lactosylceramide [LacCer]) or oligosaccharides (ex, GM1, GM3) (for a review, see [2]). The importance of orientation of headgroups in membrane lipid assembly is underlined by the very distinct properties between fluorescent derivatives of the natural D-*e*-LacCer and of L-*t*-LacCer, a compound of artificial stereochemistry [3].

SLs and cholesterol may spontaneously cluster together with glycosylphosphatidylinositol (GPI)-anchored proteins into discrete L_o_ “lipid rafts” floating among the L_d_-phase, thereby creating lateral asymmetry, i.e. representing a first deviation from the original Singer-Nicholson model [4], [5], [6], [7]. After a long controversy on their natural occurrence, lipid rafts are now widely recognized as heterogeneous entities (for reviews, see [8], [9]), that can be stabilized on their cytosolic side by multipalmitoylated proteins such as caveolins to form essentially immobile homogeneous omega-shaped invaginations, the caveolae, and transverse tubules deeply penetrating muscle cells. However, it appears that most rafts are unstable nanometric assemblies, which can only be vizualized at the plasma membrane (PM) by atomic force microscopy [10], by stimulated emission depletion far-field fluorescence nanoscopy [11], or by confocal microscopy upon cross-linking procedures, such as binding of the pentavalent B subunit of cholera toxin (CTXB) to GM1 [12] or bivalent antibody-mediated cross-linking of GPI-anchored proteins into caveolae. Attempts to visualize lipid rafts by confocal microscopy without cross-linking have consistently remained unsuccessful (for reviews, see [13], [14]). Rafts can differentially recruit mono- or di-acyled non-receptor tyrosine kinases of the Src family that regulate various signalling events [15], including at the immunological synapse [16]. PM lipid rafts also participate in clathrin-independent endocytosis [17].

Whereas a consensus on nanometric rafts has thus emerged [18], [19], the concomitant occurrence of micrometric domains at the PM of living cells under physiological conditions is debated [20], if not dismissed [2]. Their existence was originally inferred by the comparison of lateral diffusion parameters after insertion of the fluorescent PC analog, NBD-PC, based on differential Fluorescence Recovery After Photobleaching (FRAP) fields of increasing size on living fibroblasts, but direct visual evidence was less convincing at that time [21]. The first morphological evidence was provided by mixing combinations of fluorescent lipid analogs (or lipidomimetic dyes) when generating model membranes of simplified composition (liposomes) - or even derived from the more complex PM composition - yielding clear-cut, micrometric partitioning between L_o_- and L_d_-phases, with abrupt linear boundaries [22], [23], [24]. Surprisingly, secondary insertion into erythrocyte ghosts of fluorescent analogs for glycerophospholipids (expected to partition as a continuous diffuse phase) strongly labelled one or two micrometric “domains”, with distributions depending on the headgroup, but no clear equivalent was originally reported in living erythrocytes [25]. Later on, discrete micrometric patchy labelling upon insertion of other fluorescent lipid analogs into living cells was reported, but only after major lipid alterations, such as extensive surface digestion by the combination of phospholipase C with sphingomyelinase [26], or strong cholesterol depletion [27]. It is no surprise that these very artificial conditions required for micrometric segregation of tail-grafted fluorescent lipid analogs or totally artificial lipophilic fluorescent compounds favored instead the conclusion that the spontaneous formation of micrometric membrane domains of endogenous lipids in untreated living cells would be actually prevented, e.g. by interference from membrane proteins or by actin-driven membrane tension [24].

However, micrometric domains can clearly be seen by vital confocal imaging of untreated cells: living erythrocytes [28], [29], [30], [31], blood platelets [32] and nucleated cells [3], [27], [31], [33]. After careful exclusion of a variety of possible artefacts, we recently reported that three different SM analogs labelled micrometric domains at the PM of erythocytes and CHO cells, that segregated from a D-*e*-LacCer analog (yet built up on the same fluorescent ceramide backbone), thus depending on their distinctive polar headgroups. FRAP analyses confirmed different lateral diffusion properties between SM and D-*e*-LacCer analogs. The selective abrogation of BODIPY-SM domains by modulation of endogenous SM levels raised the possibility that the SM fluorescent derivative could reflect some natural large-scale compartmentation of endogenous SM [31]. Since this would represent a second revision of the Singer-Nicholson model (besides nanometric rafts), it was obviously essential to further put the micrometric compartmentation model into test.

We have since pursued two lines of investigations. Firstly, since various nanometric L_o_-rafts may coexist within a L_d_-phase, we tested how general the concept of micrometric compartmentation could be, by extending our analysis to other SL analogs as well as to tentative representatives of the L_d_-phase, while paying particular attention to the effect of temperature on phase behaviour, with important differences being noted between 30°C and 37°C. This task has been largely achieved as shown by the present report, where we evidence at least three different fluorescent lipid phases of differential order, each reaching up to 5-fold differential enrichment, covering together up to 70% of the cell surface, and depending on endogenous SM and GSLs composition. As a second line of investigations, the role of cholesterol and membrane tension will be the topic of another report (D′auria et al, in preparation).

## Results

### Experimental strategy: selection of lipid core moieties and fluorophores

Lipid core moieties and fluorophores were selected as follows. PC was studied as the most abundant glycerophospholipid representative, since it accounts for >50% of all phospholipids [2] and is the most enriched one in the outer PM leaflet, at least in erythrocytes. Thus, various fluorescent PC analogs can be readily inserted and analyzed without flip-flop nor diffusion into the cytosol of erythrocytes [34] and some, but not all, cell lines, allowing to study their organization by confocal microscopy and their dynamics by FRAP [21]. Analogs of SM were compared to those of GlcCer, D-*e*-LacCer, two major representatives of the small neutral GSLs (or cerebrosides), and GM1, one representative of gangliosides, all restricted to the outer PM leaflet. GSLs offer two experimental advantages. First, they exhibit much higher melting transition temperature (Tm) as compared with SM and PC [8], [35], [36], a very useful property to exploit temperature for investigation of phase separation and domain (de)formation in complex cellular membranes. As expected from their high-Tm values, GSLs tend to segregate from the low-Tm phospholipids in mixed membranes. As a consequence, GSL-enriched domains have a tight lateral packing density [9], which can be probed by spectral shift of emitted light at high fluorescent lipid concentration (excimers). Second, properties of the BODIPY derivative of D-*e*-LacCer (the natural compound) are very different from those of BODIPY-L-*t*-LacCer (note the artificial stereochemistry), which can serve as negative control [3]. In particular, BODIPY-D-*e*-LacCer forms excimers while BODIPY-L-*t*-LacCer does not.

To follow lipids by imaging and FRAP, we used two well-established fluorophores grafted to replace the corresponding space of alkyl tails of GSLs and PC: (i) BODIPY (boron dipyrromethenedifluoride) green (λem 505 nm; BODIPY^505^, referred to BODIPY) for its high quantum yield and photostability, that we compared to a red spectral variant we synthesized ourselves (λem 589 nm; BODIPY^589^); and (ii) the other widely used, yet more photolabile, NBD (7-nitrobenz-2-oxa-1,3-diazol-4-yl) [31], [37], [38]. For structures, see Fig. S1 We thus compared various analogs of PC, differing by the length and saturation of the non-substituted fatty acid chain ([C16:0] *vs* [C18:1]), *vs* two GSLs with a natural stereochemistry: GlcCer and D-*e*-LacCer, for which the artificial (L-*t*) stereoisomer (BODIPY-L-*t*-LacCer) served as control [3]. Fluorescent lipid analogs were mixed with equimolar defatted-BSA as carrier, cleaned of aggregates by centrifugation and generally used at 1 µM. Under our conditions, BODIPY-lipids compartmentation in the PM could be studied at trace levels in comparison with endogenous lipids (data not shown and [31]). Moreover, we checked that increasing BODIPY-SM concentration between 0.5 µM to 3 µM changed neither the number nor the size of BODIPY-SM micrometric domains in erythrocytes (data not shown). For double labelling, BODIPY^505^-tracers were compared with BODIPY^589^-analogs and with a glycosylphosphatidylinositol-anchored protein, GPI-mRFP [39], as a totally unrelated reporter of outer leaflet L_o_-phases, in which the fluorophore is not embedded within the bilayer but stands out as the bulky red fluorescent protein.

### In living erythrocytes, three different PC analogs concentrate at micrometric patches with rounded contours and sharp boundaries, controlled by temperature

**Figure 1:**
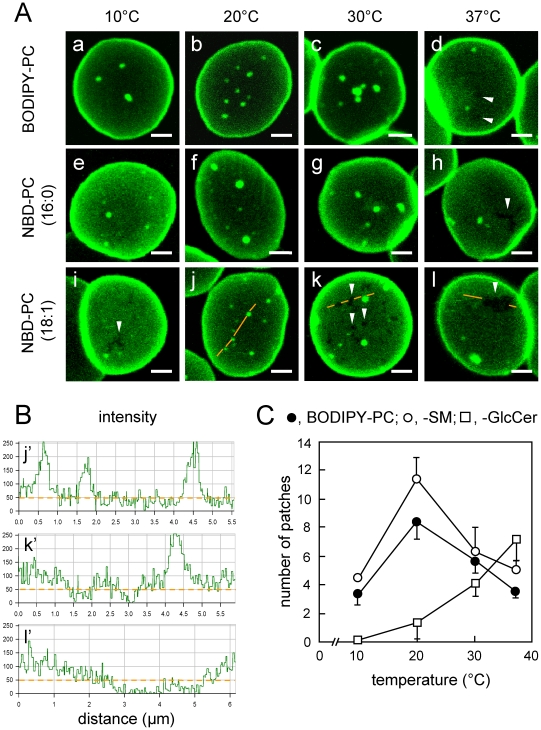
In living erythrocytes, three PC analogs concentrate at similar micrometric patches with similar temperature dependence. **(A) Confocal imaging.** Freshly isolated erythrocytes were immobilized onto poly-L-lysine-coated coverslips, labelled with the PC analogs indicated at left, washed and examined upside-down by confocal microscopy (i.e. with the free erythrocyte surface close to the objective) at the temperatures indicated above. Notice rounded areas of strong concentration (brilliant patches) over a weak diffuse labelling. Non-labelled foci with less regular contours (arrowheads) are also visible with all tracers at 37°C (d,h,l) and are best evidenced with NBD-PC (18∶1) at all temperatures (i-l). All scale bars, 2 µm. **(B) Relative concentration** **.** Fluorescence enrichment and exclusion are illustrated for NBD-PC (18∶1) by line intensity profiles along the paths indicated by orange lines at panels A, j-l, with background set at zero outside cells (a reference level confirmed by non-labelled foci). Lines are interrupted to better evidence foci of concentration or exclusion. **(C) Morphometry: effect of temperature and comparison with SL analogs.** Number of patches labelled by BODIPY-PC (closed circles), -SM (open circles) or -GlcCer (open squares) was recorded per hemi-cell surface at the indicated temperatures (means±SEM of 3-7 independent experiments, each with 3–45 cells, except at 10°C for BODIPY-SM and -GlcCer, 1 experiment). Notice the opposite response to temperature increase between BODIPY-PC and -SM *vs* -GlcCer.

We reported last year that insertion of BODIPY-SM or -GlcCer at the outer membrane of freshly isolated, adherent erythrocytes generates brightly fluorescent micrometric domains, showing up to ∼8-fold enrichment over the bulk of the membrane, yet with a different temperature optimum (20°C and 37°C, respectively) [31]. As shown by Fig. 1A, BODIPY-PC, NBD-PC with a short saturated alkyl chain (16∶0) or NBD-PC with a longer, mono-unsaturated alkyl chain (18∶1) also revealed multiple well-defined brilliant rounded patches of micrometric size (∼0.5 µm in diameter). Brilliant patches were undistinguishable between tracers. The rest of the cell surface appeared weakly and homogenously labelled, except at discrete foci that seemed to exclude the tracer (“moth-eaten pattern”, arrowheads), best visible with the unsaturated PC analog, arguably the closest to natural PC. Quantitation by line intensity profiles revealed that patches were enriched up to ∼5-fold over the rest of the PM (exemplified at Fig. 1B for NBD-PC [18∶1], selected to best reveal non-labelled foci). This quantitation confirmed the homogeneity of diffuse baseline (∼50 a.u.) and patchy labelling (most being slightly below 250 a.u.). Very much like for BODIPY-SM, the average number of BODIPY-PC patches varied significantly with temperature (Fig. 1C), increasing from ∼3 at 10°C, peaking to ∼8 at 20°C, then declining back to ∼3 at 37°C, to vanish >40°C (data not shown). The common behaviour of BODIPY-PC and -SM was in sharp contrast to that of BODIPY-GlcCer (only differing from the previous one by the polar headgroup), which did not show detectable patches at 10°C, but a steady increase number with temperature. When the entire procedure for erythrocytes labelling, washing and imaging of BODIPY-GlcCer was conducted at 37°C, rounded patches of micrometric size were undistinguishable, indicating that these domains are not a metastable state due to exposure at low temperature (Fig. S2). Interestingly, micrometric domains remained immobile for at least 45 min (data not shown).

Taken together, these data indicated that all five analogs (three PC, BODIPY-SM and -GlcCer) spontaneously self-assembled on living erythrocytes. To rule out that these patches reflect artificial vesicles stuck to the erythrocyte surface or any other significant surface feature, we have manipulated domain abundance by marginal cholesterol depletion (preserving BODIPY-SM insertion but leading to BODIPY-SM domain disappearance) and membrane anchorage (by impairing 4.1R complex via PKC activation; doubling the number of BODIPY-SM domains). Yet, the surface of BODIPY-lipid-labelled erythrocytes remained featureless by scanning electron microscopy (D′Auria *et al*, in preparation).

To next examine whether these BODIPY-PC, -SM and -GlcCer micrometric patches were structurally distinct, we used the two following approaches: (i) differential excimer formation; (ii) double-labelling using the BODIPY^589^-derivatives of SM and GlcCer we had previously synthesized and successfully applied to CHO cells [31].

### In living erythrocytes, BODIPY-PC patches do not represent ordered clusters, in contrast to BODIPY-SM and -GSL micrometric domains

**Figure 2:**
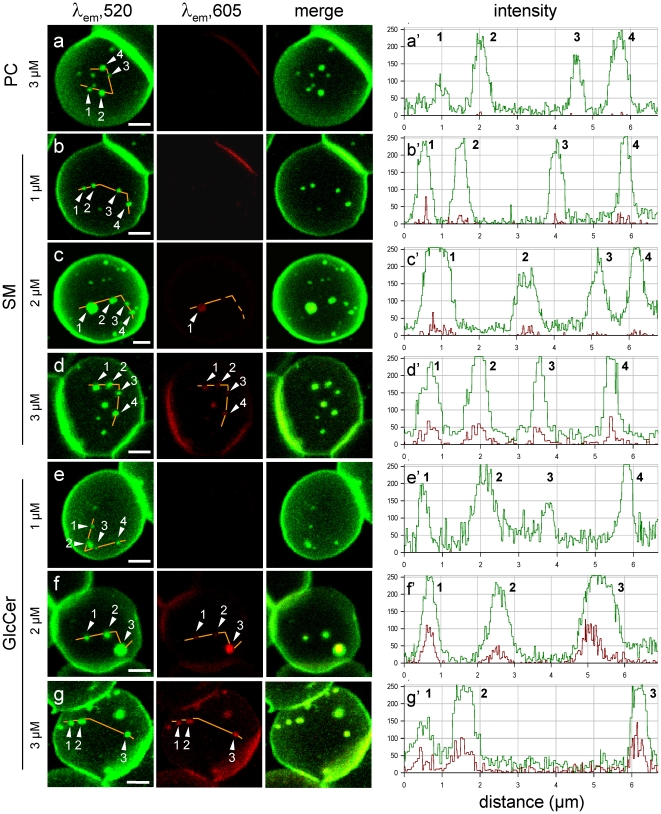
In living erythrocytes, BODIPY-SLs, but not BODIPY-PC, exhibit differential spectral shift at high concentration. **Left, confocal imaging.** Freshly isolated erythrocytes were labelled as at Fig. 1, using BODIPY-PC (a), -SM (b-d) or -GlcCer (e-g) at 1 µM (b,e), 2 µM (c,f) or 3 µM (a,d,g), washed and immediately examined by confocal microscopy at 20°C (a-d) or 37°C (e-g). Images were all generated with λexc 488 nm, with simultaneous recording in the green (left; λem 520 nm) and red channels (middle; λem 605 nm), then merged (right). Note that yellow signal in merged images, indicative of ordered clustering (excimers), is essentially absent at 3 µM for BODIPY-PC, weak for -SM and strong for -GlcCer. All scale bars, 2 µm. **Right, quantitation of conventional and excimer emission.** Intensity profiles were recorded along the paths indicated by the continuous orange lines at left; due to different settings, the minimal baseline values cannot be compared with other figures. Numbers #1-4 refer to the indicated patches. Average red/green emission ratio for BODIPY-SM is <20% at 3 µM (d′), but already >30% for BODIPY-GlcCer at 2 µM (f′).

**Figure 3:**
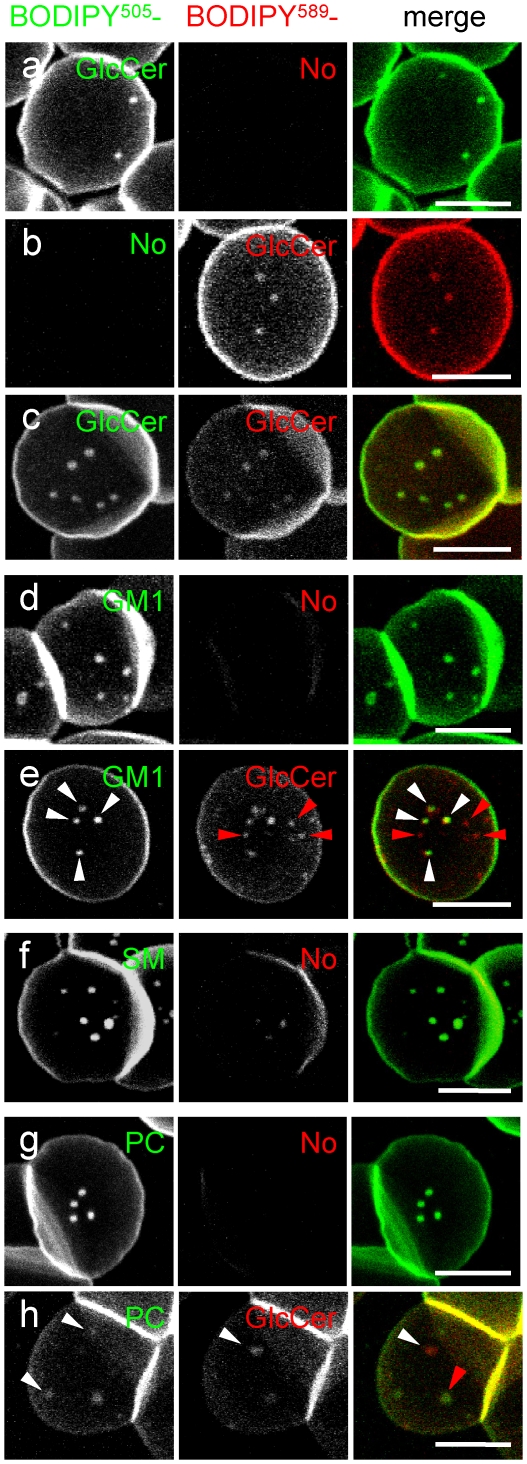
In living erythrocytes, micrometric domains of BODIPY-GlcCer co-localize with -GM1, but not with -PC. Erythrocytes were labelled either with only one of the indicated BODIPY^505^-lipids (1.5 µM -GlcCer and -PC; 1.3 µM -GM1; 1 µM -SM [a,g,d,f]), or 4 µM BODIPY^589^-GlcCer (b); or sequentially labelled with 4 µM BODIPY^589^-GlcCer then with the indicated BODIPY^505^-lipids (same concentration as above) in the continued presence of BODIPY^589^-GlcCer (c,e,h). Images were sequentially recorded in the green (left) and red (middle) channels with settings adjusted to best match signal intensities, then merged (right). Notice that no BODIPY^505^-lipids produced excimers at the concentrations used, except BODIPY-SM (f) precluding unambiguous testing of co-distribution with BODIPY^589^-GlcCer. Despite lower intensity and resolution of the red tracer, BODIPY^505^- and BODIPY^589^-GlcCer show perfect co-localization (c). At (e), all BODIPY^505^-GM1 domains also recruit BODIPY^589^-GlcCer (white arrowheads). Additional spots, only but less intensely labelled by BODIPY^589^-GlcCer (red arrowheads) reflect the higher number of BODIPY-GlcCer domains, attributed to the higher melting temperature of natural GlcCer than for GM1. In contrast, BODIPY^505^-PC and BODIPY^589^-GlcCer mostly segregate (h; white arrowheads; a rare co-localization is indicated by the red arrowhead). All scale bars, 5 µm.

The organization of BODIPY-PC, -SM and -GlcCer patches can be probed for clustering-dependent change in spectral properties, known as excimer formation. As recently reported for BODIPY-D-*e*-LacCer [3] and -SM [31], this phenomenon results in a partial shift of the primary emission peak at λem 520 nm (“green”) to a secondary emission peak at λem 605 nm (“red”), whose combination generates a yellow signal (shown at merge). We therefore looked at green and red fluorescence emission from BODIPY-PC, -SM and -GlcCer patches on erythrocytes, after insertion from 1 µM (usual concentration) to 3 µM, at the optimal temperature for patches formation (20°C for BODIPY-PC and -SM *vs* 37°C for BODIPY-GlcCer). As shown by Fig. 2, no excimer phenomenon could be detected at BODIPY-PC patches up to the highest concentration tested (Fig. 2a). For BODIPY-SM, excimer formation was barely detected by line scans at 1 µM (Fig. 2b′; see also Fig. 3f), became more visible by direct image inspection at 2 µM (Fig. 2c), and further increased at 3 µM (Fig. 2d,d′), at which concentration red/green emission ratio approached, but did not exceed 20%. For BODIPY-GlcCer, excimer formation was already obvious at 2 µM and yielded emission ratios exceeding 30% (Fig. 2f-g′).

### In living erythrocytes, distinct micrometric preferential assemblies coexist

To further examine whether these micrometric patches were structurally distinct, we performed double-labelling using the BODIPY^589^-derivative of GlcCer. Despite the much lower quantum yield of the red tracer requiring much higher laser power, BODIPY^589^-GlcCer showed absolutely no bleed-through in the green channel (Fig. 3b). In addition, BODIPY^505^- and BODIPY^589^-GlcCer perfectly co-localized (Fig. 3c). As shown by the fifth row, the two GSLs, BODIPY^505^-GM1 and BODIPY^589^-GlcCer, also showed extensive co-localization. All BODIPY^505^-GM1 domains also recruited BODIPY^589^-GlcCer, but the converse was not true: additional spots were only - albeit less intensely - labelled by BODIPY^589^-GlcCer, despite the higher sensitivity of the green fluorophore. This necessarily reflects the >2-fold higher number of BODIPY-GlcCer domains (data not shown), which can be reasonably attributed to the higher melting temperature of natural cerebrosides (monophasic; between 50 and 70°C) than for GM1 (biphasic; ∼20°C and 45°C) [40]. In contrast, BODIPY^589^-GlcCer and BODIPY^505^-PC were mostly segregated (last row), as predicted from a distinct lipid order (see Fig. 2).

### In CHO cells, micrometric domains labelled by BODIPY-SM, -GSL, -PC and -L-*t*-LacCer also differed in intrinsic ordering

**Figure 4:**
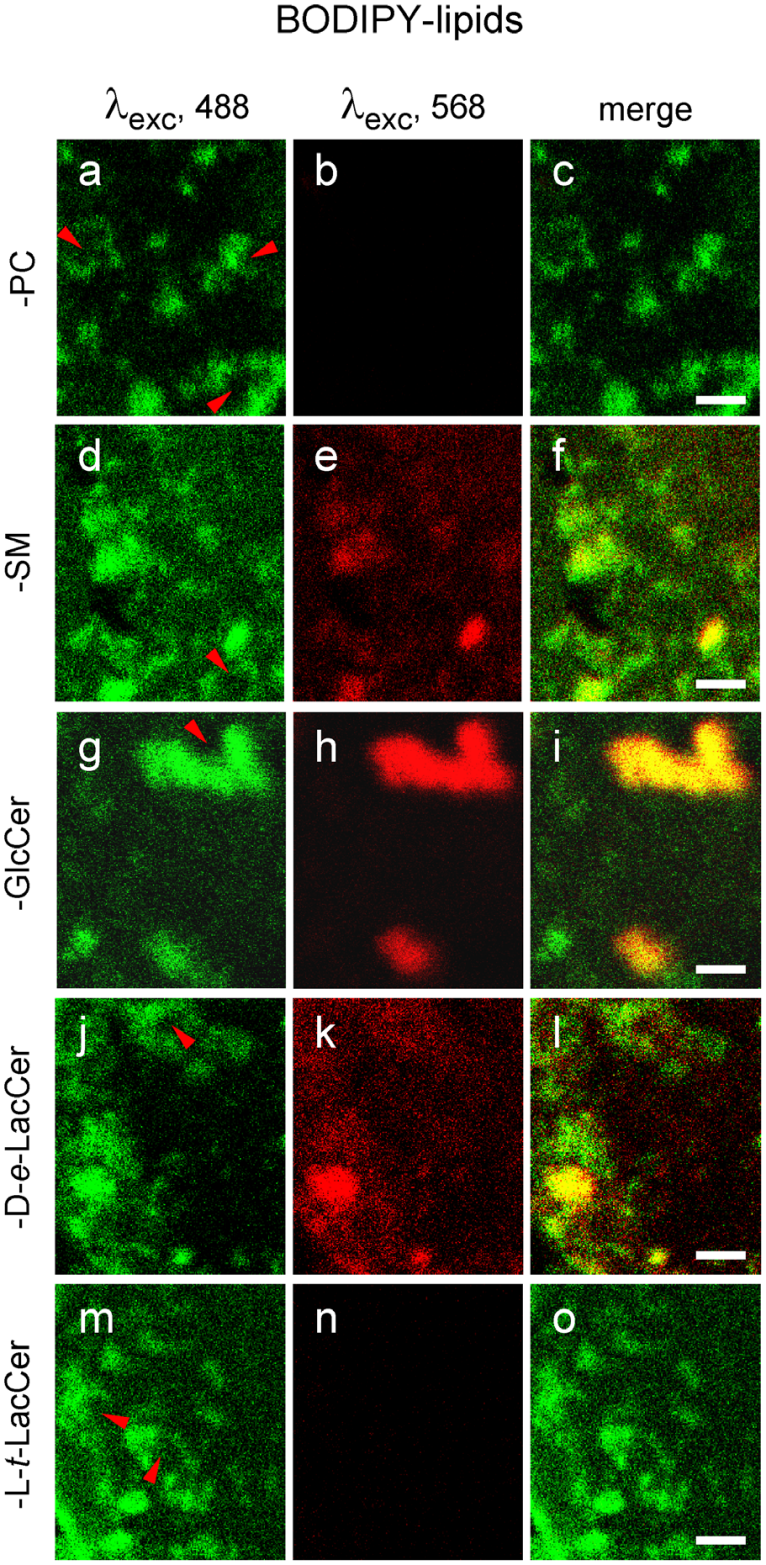
In CHO cells, only BODIPY-D-*e*-SLs form micrometric patches competent for excimer formation. CHO cells were surface-labelled at low temperature with 5 µM of the indicated BODIPY-lipids, washed and immediately examined by confocal microscopy at 10°C to prevent endocytosis. Bottom confocal sections are shown. Images were recorded in the green channel (left) at the usual intensity, then in the red channel at 30-times higher laser power (middle) and merged (right). Whereas excimer formation (yellow signal at right) is obvious for BODIPY-SM (f) and BODIPY-GSLs with natural stereochemistry [-GlcCer (i) and -D-*e*-LacCer (l)], no spectral shift is observed using BODIPY-PC (c) and a GSL with artificial stereochemistry, -L-*t*-LacCer (o). For the two latter derivatives, notice convoluted wavy labelling, with notches indicated by red arrowheads. All scale bars, 2 µm.

Since erythrocytes are very special cells, we extended our investigations to CHO cells, where BODIPY-SM micrometric domains were observed and shown to form excimers [31]. BODIPY-PC and -D-*e*-LacCer superficial patches were readily detected on CHO cells as well. As shown by resistance to K^+^-depletion (Fig. S3b,e) and latrunculin B treatment (Fig. S3c,f), these patches genuinely reflected packaging at the PM, and not peripheral endosomes [41] nor actin-dependent surface extensions. To sensitize excimer formation in CHO cells, BODIPY-lipids were inserted at a higher concentration (5 µM). As shown by Fig. 4b,c,n,o, BODIPY-PC and -L-*t*-LacCer micrometric patches were incompetent to form excimers, in contrast to BODIPY-SM (Fig. 4e,f), and especially -D-*e*-LacCer (Fig. 4k,l) as well as -GlcCer (Fig. 4h,i). These data indicated that, in both cell types, micrometric domains labelled by lipid analogs differed in intrinsic ordering, with the following ranking: BODIPY-GSLs > -SM > -PC or -L-*t*-LacCer.

To further distinguish three potentially distinct micrometric membrane domains types in CHO cells, we combined the following approaches: (i) differential modulation of individual domain structure by temperature; (ii) spatial complementarity; (iii) comparison to an unrelated reporter of outer PM leaflet L_o_-domains, GPI-mRFP; (iv) differential modulation by endogenous SLs depletion; as well as (v) differential dynamics by FRAP and its differential modulation by manipulation of endogenous SLs and by temperature.

### Peak concentrations and domain boundaries of BODIPY-PC and -L-*t*-LacCer *vs* -D-*e*-LacCer in CHO cells show opposite temperature dependence

**Figure 5:**
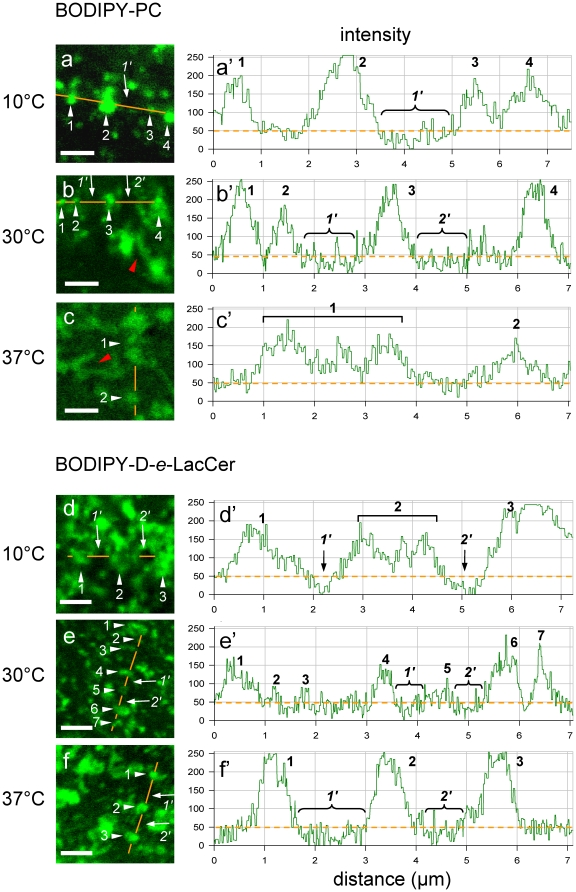
Differential effect of temperature on boundaries of BODIPY-PC and -GSL micrometric domains in CHO cells. CHO cells were surface-labelled at 4°C with 1 µM BODIPY-PC (a-c) or -D-*e*-LacCer (d-f), washed and transferred to the indicated temperatures, at which the bottom cell surface was immediately imaged. **At left (a-f), confocal imaging.** Notice convoluted labelling for BODIPY-PC at 30°C and 37°C, with notches indicated by red arrowheads. All scale bars, 2 µm. **At right (a′-f′), quantitation of relative concentrations** by line intensity profiles (orange lines at left). Individual, well-defined peaks above fluorescence “baseline” (orange dotted lines at the level of 50 a.u. at right) are numbered from #1 up to 7; clustered patches are indicated by straight brackets; foci below baseline are numbered from #1′ to 2′, and indicated by rounded brackets. Notice that BODIPY-PC concentrates up to 30°C into sharp peaks which vanish at 37°C, whereas BODIPY-D-*e*-LacCer sharp peaks are best defined at 37°C.

We first reasoned that segregation of domains should somehow be reflected at their boundaries. This was examined after a very brief warming up, from 10°C to 37°C, to minimize internalization (typically within 2 min). BODIPY-PC formed in CHO cells sharply-defined micrometric domains from 10°C to 30°C (Fig. 5a-b′), which showed a comparable, up to ∼5-fold enrichment over the “rest” of the membrane, as in erythrocytes (orange dotted line in intensity profiles at a′,b′), and areas of local depletion could also be clearly seen (arrows at left; continuous pixels below baseline under rounded brackets at right). In contrast, at 37°C, BODIPY-PC boundaries became fuzzy and domains were both less concentrated and much more elongated and indented (Fig. 5c,c′). The opposite temperature effect was observed for the L_o_-BODIPY-D-*e*-LacCer, showing large domains with fuzzy boundaries at 10°C (Fig. 5d,d′) and most obvious peaks of concentration contrasting with foci of local exclusion at 30°C and 37°C (Fig. 5e-f′). As shown at Fig. S4a, BODIPY-L-*t*-LacCer also concentrated at 10°C into sharply-defined micrometric domains, like BODIPY-PC at this temperature. Incidentally, mammalian cells are not all (or always) operating at 37°C in the body, even without fever or severe hypothermia: for example ∼30°C is a physiological temperature for human erythrocytes when circulating across capillaries of nose mucosa or peripheral skin, as everybody may have experienced.

Together, these data indicated that the two L_d_-tracers, BODIPY-PC and -L-*t*-LacCer, concentrated on the CHO cell surface at low temperatures into well-defined, mostly rounded domains, but spread more broadly at the physiological temperature into more elongated, irregular and interconnected regions with lesser intensity and more fuzzy boundaries; the converse was found for L_o_-GSL analogs with a natural stereochemistry. This opposite modulation of individual domain structure by temperature provided a first functional evidence that L_d_- and L_o_-tracers formed distinct micrometric domains.

### BODIPY-PC, -SM and -GlcCer labelling of CHO cell surface is complementary, thus additive

**Figure 6:**
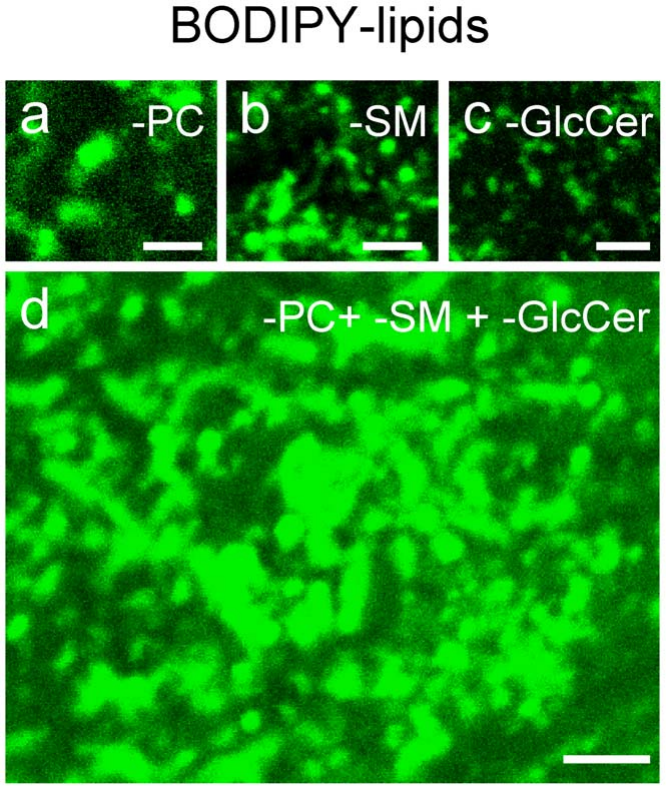
Complementarity between BODIPY-PC, -SM and -GSL domains. Cells were surface-labelled at 4°C with BODIPY-PC (a,d), -SM (b,d) and -GlcCer (c,d, 1 µM each), either alone (a-c) or combined (d). After washing, the bottom cell surface was immediately imaged by confocal microscopy at 20°C to maximize domain formation for each component. All scale bars, 2 µm. Notice that BODIPY-PC, -SM and -GlcCer patches separately label ∼25% of the CHO cell surface at a-c, whereas simultaneous labelling covers ∼70%.

As a second approach to support the coexistence of several micrometric domains, we looked for complementarity. Cumulative areas occupied by BODIPY^505^-PC, -SM and -GlcCer micrometric patches, either alone or combined, were estimated after surface labelling at 4°C, then rapid imaging after transfer at 20°C, as a compromise for simultaneous optimal patchy labelling by each analog (see Fig. 1C). The fractional coverage of the CHO bottom PM by the discrete micrometric patches labelled by 1 µM BODIPY^505^-PC, -SM or -GlcCer alone was very similar (∼ 25% each; Fig. 6a-c), and did not expand at increasing concentration (see above; also compare Fig. 6a, recorded at 1 µM, *vs* Fig. 4a at 5 µM). Since double-labelling using two pairs of spectral variants were mutually exclusive [31], one could thus expect that triple addition would cover most of the CHO bottom cell surface. As shown by Fig. 6d, simultaneous labelling by the three tracers indeed covered ∼70% of this surface, still with a predominantly patchy pattern arguing against a mere coalescence and indicating instead complementarity as in a jigsaw puzzle. Similar additivity was observed at 10°C (data not shown). This type of result ruled out interference due to the different structure between the two BODIPY spectral variants (see Fig. S1) and provided a second morphological line of evidence in favour of the coexistence of distinct, non-overlapping BODIPY-lipid domains.

### BODIPY-PC, -SM and -GSL differentially segregate from a GPI-anchored red fluorescent protein reporter

**Figure 7:**
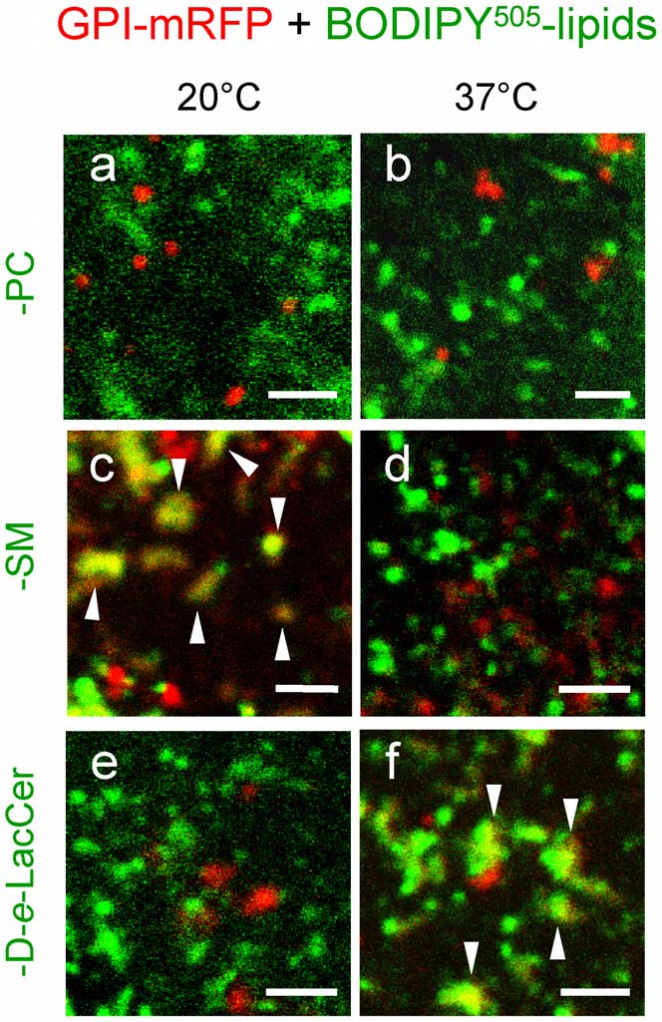
Differential segregation of BODIPY-PC, -SM and -GSL from a GPI-anchored fluorescent reporter on CHO cells. One day after transfection with an expression vector for the L_o_-glycosylphosphatidylinositol-anchored protein reporter, GPI-mRFP (red), CHO cells were transferred to 20°C (left; a,c,e) or 37°C (right; b,d,f), labelled with BODIPY^505^-PC (a,b), -SM (c,d), or -D-*e*-LacCer (e,f), washed and immediately analyzed in the green then in red channel. Notice that the GPI-reporter labels micrometric patches (red) showing extensive colocalization with BODIPY-SM at 20°C but not at 37°C, and with BODIPY-D-*e*-LacCer at 37°C but not at 20°C. For separated (single-channel) imaging with BODIPY^505^-SM and -D-*e*-LacCer, see Fig. S5.

A third alternative approach was to use for reference a totally unrelated lipid probe emitting red fluorescence, based on a fully natural lipid moiety and a fluorophore entirely located outside the bilayer. For this purpose, CHO cells were transfected with an expression vector for the GPI-anchored monomeric red fluorescent protein reporter (GPI-mRFP), shown by Simons and his colleagues to be restricted to L_o_-phase(s) [39]. In addition, in view of the distinct temperature-dependence exhibited by BODIPY-lipid assemblies on both erythrocytes (Fig. 1) and CHO cells (Fig. 5), we systematically examined GPI-mRFP-expressing cells for co-localization with each representative BODIPY-lipid at 20°C and 30°C *vs* 37°C. As can be seen at Fig. 7, GPI-mRFP readily formed micrometric patches at the CHO cell surface one day after transfection, without noticeable difference from 20°C to 37°C (compare red spots at Fig. 7a,c,e *vs* b,d,f). BODIPY^505^-PC assemblies essentially segregated from GPI-mRFP at both temperatures (green patches at Fig. 7a,b). In contrast, BODIPY-SM and -D-*e*-LacCer domains showed a strong, yet distinct temperature-dependent, association with the GPI reporter (Fig. 7 and Fig. S5). At 20°C, BODIPY-SM micrometric domains mostly co-segregated with GPI-mRFP, but this association decreased upon transfer to 30°C (Fig. S5) and had vanished at 37°C (compare Fig. 7c, arrowheads, *vs* 7d; Fig. S5). Conversely, BODIPY-D-*e*-LacCer domains showed no co-localization with GPI-mRFP at 20°C, little association at 30°C and strong co-localization at 37°C (compare Fig. 7e *vs* 7f, arrowheads; Fig. S5).

These data thus confirmed that three biochemically distinct types of micrometric lipid compartments co-existed at the cell surface. Moreover, they further suggested that the differential association of BODIPY-lipids with the L_o_-GPI reporter actually reflected intrinsic BODIPY-lipid ordering. Indeed, there was no association with BODIPY-PC which also did not generate excimers (see Fig. 2a,a′), indicating that, like endogenous PC, its BODIPY analog behaves as a L_d_-tracer. For the two analogs of the L_o_-tracers, BODIPY-SM and -GlcCer (see Fig. 2d,d′ and g,g′), association with GPI-mRFP occurred at the optimal temperature for domain formation on erythrocytes (i.e. 20°C for BODIPY-SM *vs* 37°C for -GlcCer, see Fig. 1C) and at an increasing temperature with increasing excimer propensity.

### BODIPY-PC and -L-*t*-LacCer *vs* -SM micrometric domains are differentially affected by endogenous SLs depletion

To tackle the relation between the fluorescent micrometric assemblies labelled by insertion at tracer levels of the various lipid analogs with their endogenous lipid counterparts, we next tested the effects of selective depletion for endogenous GSLs, known to stabilize L_o_-domains [42]. To this aim, CHO cells were first treated with the GlcCer synthase inhibitor, D-PDMP (D-threo-1-phenyl-2-decanoylamino-3-morpholino-1-propanol) [43]. As documented by Table S1, this treatment caused a ∼60% loss of GlcCer and a ∼45% loss of GM3, the major GSL in this cell line [44]. These effects were lipid-specific, since D-PDMP did not alter the SM level, and drug-specific, since its inactive stereoisomer, L-PDMP, did not appreciably modify GSLs levels (data not shown). Alternatively, CHO cells were treated with bacterial sphingomyelinase (SMase) [45], which led to a ∼50% depletion of endogenous SM, without any effect on GlcCer levels, as expected. Combined depletion was achieved by the dihydroceramide synthase inhibitor, fumonisin B1 (FB1).

**Figure 8:**
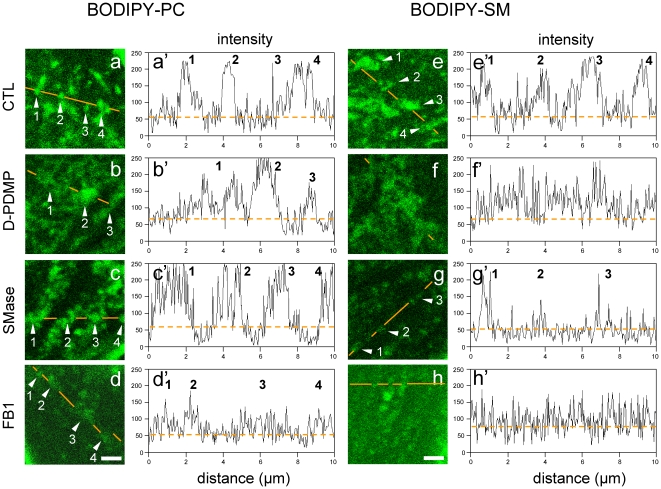
BODIPY-PC and -SM enriched micrometric domains show differential sensitivity to endogenous GSL and SM depletion. CHO cells were either kept untreated (a,e; CTL); selectively depleted for GSLs with the GlcCer synthase inhibitor, D-PDMP (b,f) or sphingomyelin with sphingomyelinase (SMase; c,g); or depleted of both using the upstream, dihydroceramide synthase inhibitor, fumonisin B1 (FB1; d,h), then surface-labelled with BODIPY-PC (a-d) or -SM (e-h), washed and immediately examined by confocal microscopy at 10°C. All images are bottom confocal sections recorded at the same laser power and magnification (scale bars, 2 µm). For each panel, intensity profiles along paths indicated by orange lines on confocal images are shown at right (a′-h′), by reference to baseline homogenous labelling (∼50 a.u.; horizontal dotted lines). Notice in control cells similar well-defined patches for the PC and the SM analogs (a,e) with individual sharp peaks (arrowheads #1-4). Most BODIPY-PC micrometric patches/peaks resist GSL depletion by D-PDMP (b) or SM depletion by SMase (c). In contrast, essentially all well-defined micrometric BODIPY-SM patches vanish upon either D-PDMP (f) or SMase (g). For similar properties between BODIPY-PC and -L-*t*-LacCer, see Fig. S4.

As shown by confocal microscopy (Fig. 8b,b′,c,c′; Fig. S4b,c), well-defined patches of BODIPY-PC and -L-*t*-LacCer could still be observed in either D-PDMP- or SMase-treated CHO cells, although average peak intensity declined and boundaries were more irregular and less sharp (see line intensity profiles at Fig. 8b′c′). The dependence of BODIPY-PC and -L-*t*-LacCer micrometric assemblies for either endogenous GSLs and SM was confirmed by their disappearance upon combined depletion of GSLs and SM by FB1 treatment (Fig. 8d,d′; Fig. S4d), as if a L_d_-phase required only exclusion by a L_o_-phase. In contrast, BODIPY-SM domains were strongly impaired by D-PDMP (Fig. 8f,f′), and almost abrogated by SMase (Fig. 8g,g′), suggesting that BODIPY-SM could somehow reflect compartmentation of endogenous SM, and as if BODIPY-SM domains would further depend of a subcompartmentation of the L_o_-phase by the more ordered endogenous GSLs.

Altogether, confocal imaging qualitatively indicated that BODIPY-PC and -L-*t*-LacCer *vs* -SM assemblies could be differentially modulated by the level of endogenous SLs and confirmed that BODIPY-SM domains critically depended on endogenous SM, thus providing the first evidence for differential association of the three types of BODIPY-lipid assemblies with endogenous lipids.

### PC- and L-*t*-LacCer-, but not D-*e*-GSL-analogs show strong restriction to lateral diffusion in large photobleached fields at 30°C

**Figure 9:**
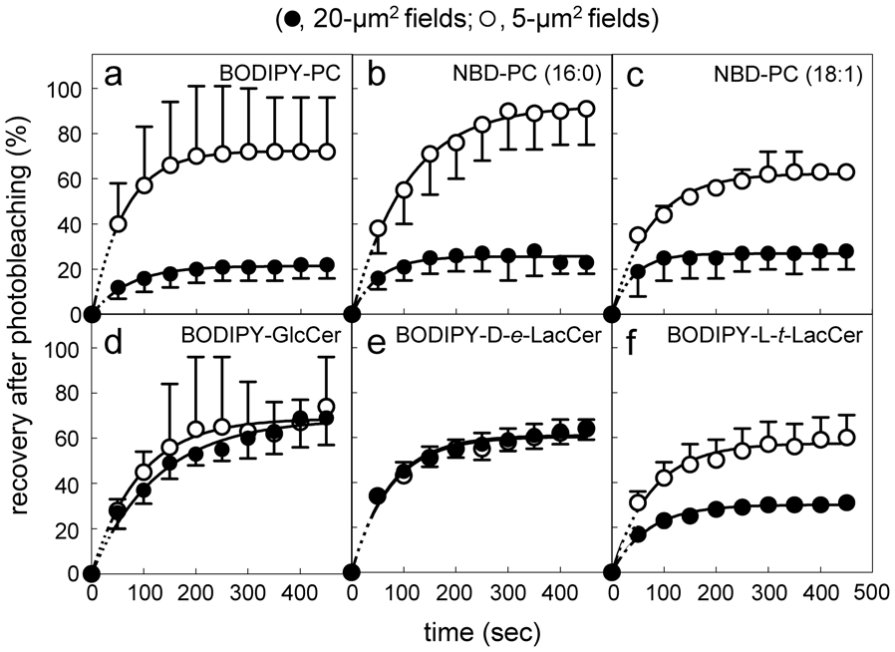
PC and L-*t*-LacCer analogs show the most severe restriction to lateral diffusion at 30°C. CHO cells were surface-labelled with the indicated BODIPY- or NBD-PC analogs (a, BODIPY-PC; b, NBD-PC [16∶0]; c, NBD-PC [18∶1]), or BODIPY-GSL analogs with either a natural stereochemistry (d, -GlcCer; e, -D-*e*-LacCer) or artificial stereochemistry (f, -L-*t*-LacCer), rapidly washed and immediately analyzed by FRAP at 30°C in fields of either 20 µm^2^ (filled symbols) or 5 µm^2^ (open symbols), using a Bio-Rad confocal microscope. Fluorescence recovery is expressed as percentage of signal before photobleaching, after correction for residual fluorescence immediately after bleaching. Values are means±SEM of 3-to-112 experiments and are fitted to monoexponential functions. Data for BODIPY-D-*e*-LacCer are reproduced from [31], for comparison purposes. Notice that lateral diffusion of PC analogs and BODIPY-L-*t*-LacCer is restricted when analyzed in large fields as compared with small fields (a-c; f), contrasting with undistinguishable mobility of D-*e*-GSL analogs in the two field sizes (d,e).

The differential effect of temperature on BODIPY-PC *vs* GSLs micrometric domains at the cell surface of erythrocytes and CHO cells pointed to a *thermodynamic effect*, involving different phase behaviour and predicting different lateral mobility properties. The latter was tested by FRAP, based on the systematic comparison of recovery after photobleaching areas of respectively 5 µm^2^ (referred to as “small fields”) and 20 µm^2^ (“large fields”) [31]. This analysis is shown at Fig. 9 and mobile fraction values at infinite time of recovery are compiled in Table S2. Extensive fluorescence recovery was observed for BODIPY-GlcCer and -D-*e*-LacCer, irrespective of photobleaching small or large fields, reaching an average mobile fraction of ∼65% (Fig. 9d,e). Similar results were observed for NBD-GlcCer (see Table S2). In contrast, fluorescence recovery of the three PC analogs at 30°C levelled off at ∼30% in 20-µm^2^ fields (Fig. 9a-c, closed symbols) but this strong restriction was largely relaxed in 5-µm^2^ fields (mobile fraction, ∼65%; Fig. 9a-c, open symbols). BODIPY-PC properties using FRAP were again mimicked by the artificial BODIPY-L-*t*-LacCer (Fig. 9f). Since we recently reported a partial, yet very consistent restriction for BODIPY-SM [31], comparative FRAP properties in large *vs* small fields allows one to independently rank BODIPY-lipid dynamics in large fields as follows: BODIPY-GSLs > -SM > -PC or -L-*t*-LacCer, providing a thermodynamic justification for their assignment to different assemblies.

### Transfer to 37°C largely relaxes restriction to BODIPY-PC lateral diffusion in large fields

**Figure 10:**
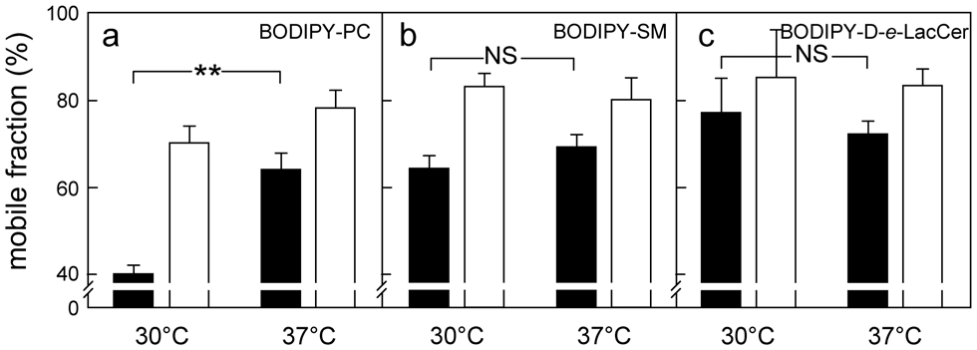
BODIPY-PC lateral diffusion in large fields is selectively restricted at 30°C but largely relaxed at 37°C. CHO cells were surface-labelled with BODIPY-PC (a), -SM (b) or -D-*e*-LacCer (c), rapidly washed and analyzed by FRAP immediately after transfer to 30°C or 37°C, in either 20-µm^2^ (filled bars) or 5-µm^2^ fields (open bars), using the Zeiss LSM510 confocal microscope. Mobile fractions are means±SEM of 4-to-32 experiments. N.S., not significant; **, p<0.01.

As shown by Fig. 5, BODIPY-PC confocal imaging had revealed a major difference between 30°C (discrete assemblies with sharp boundaries by line intensity scans) and 37°C (looser assemblies and boundaries). This suggested that BODIPY-PC dispersion at 37°C would *ipso facto* relax its selective restriction to lateral diffusion in large fields at ≤30°C. This was confirmed by comparing FRAP properties at these two temperatures: the strong difference in mobile fraction between BODIPY-PC *vs* -SLs observed after photobleaching large fields at 30°C was largely decreased at 37°C (Fig. 10), thereby providing a thermodynamic explanation for the differential domain stability in untreated cells, despite moderate temperature change.

### Restriction to BODIPY-PC lateral diffusion in large fields at 30°C depends on endogenous SLs

**Figure 11:**
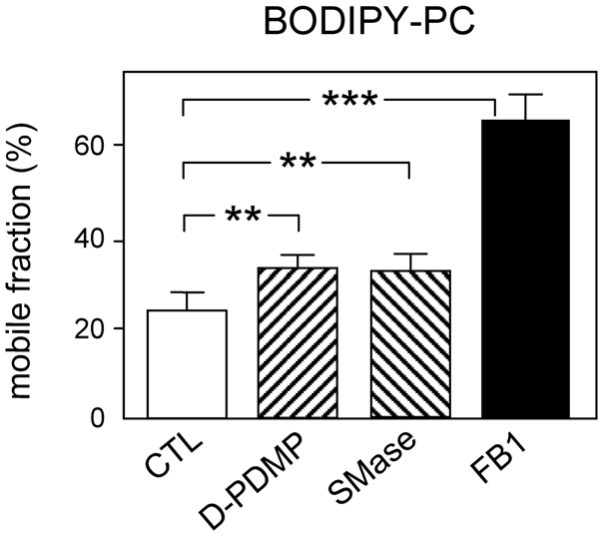
Restriction to BODIPY-PC large-scale lateral diffusion at 30°C depends on both endogenous GSLs and SM. CHO cells were either kept untreated (open bars); or treated with D-PDMP (ascending striped bar), SMase (descending striped bar) or FB1 (filled bar). For efficiency of depletion, see Table S1. Cells were then surface-labelled with BODIPY-PC, washed and rapidly transferred to 30°C for immediate FRAP analysis in 20- µm^2^ fields using the Bio-Rad confocal microscope. Mobile fractions are means±SEM of 3-to-16 experiments. **, p<0.01; ***, p<0.001.

Having shown by confocal imaging that micrometric assemblies of BODIPY-PC were only partially altered upon selective depletion of either endogenous GSLs or SM, but essentially abrogated upon combined depletion by FB1 treatment (see Fig. 8a-d), we thus tested whether restriction to the lateral diffusion BODIPY-PC at 30°C would likewise depend on endogenous lipids, using mobile fractions as a direct quantitative assay. This approach had already revealed that endogenous SM depletion further restricted recovery of BODIPY-SM after FRAP in large fields, both selectively and reversibly ([31], see Fig. 11D). As shown by Fig. 11, selective depletion of GSLs by D-PDMP or SM by SMase both partially relaxed BODIPY-PC lateral diffusion, raising the mobile fraction from as low as ∼25% to ∼35%. Combined depletion by FB1 further relaxed diffusion, so that mobile fraction values (∼60%) reached those of non-restricted tracers such as BODIPY-GSLs. These data thus indicated that endogenous GSLs and SM both contribute to restrict BODIPY-PC large-scale lateral mobility.

## Discussion

### Overview

We recently reported that BODIPY- and NBD-fluorescent analogs for the two classes of L_o_-SLs, SM and GSLs, spontaneously concentrated into distinct discrete micrometric domains on erythrocytes and CHO cells, with a differential dependence for temperature, thus concluded on at least two micrometric tracer assemblies, with potential relevance for their endogenous counterparts [31]. We here extend this analysis to the same analogs for two L_d_-lipids, PC and an artificial LacCer stereoisomer, and provide several additional lines of evidence for the coexistence of at least three types of micrometric assemblies. These include: (i) conventional imaging (combinations of double-labelling; additivity of coverage); (ii) ordering (differential propensity for excimer); (iii) thermodynamics (differential effect of temperature on the assemblies and their ability to co-localize with a GPI-anchored reporter, differential lateral mobility, and differential effect of temperature on lateral mobility); and (iv) PM composition (differential effect of selective *vs* combined depletion). The latter observations raise the hypothesis that spontaneous concentration of fluorescent membrane lipids into distinct micrometric assemblies might reflect preexisting compartmentation of endogenous PM lipids into non-overlapping domains of differential order: GSLs > SM > PC, resulting into differential self-adhesion of the two former, with exclusion of the latter (segregation by default).

### Necessary caution on, but potential of, PC- and GSL-analogs as surrogate tracers for endogenous PM lipids

Although lipid analogs we selected have been widely used by a large number of laboratories, their validity as *bona fide* surrogates for their endogenous counterparts can be questioned, because substitution by fluorophores of about half of one alkyl chain deeply embedded in the bilayer likely affects their biophysical properties, even when not appreciably altering space-filling. Although we wish to stress that great caution is required before extrapolating observations on BODIPY- or NBD-analogs to their natural counterparts, our previous report comprised a considerable number of controls supporting the view that BODIPY^505^-SM can be used as a reasonable *qualitative* surrogate tracer of endogenous SM at the PM of both erythrocytes and CHO cells. Erythrocytes are minimal living cells, yet biological objects that can be easily manipulated (on which the Singer-Nicholson model was historically based). Nucleated CHO cells offer broader relevance, offer a large registry of mutant cell lines [46], and are amenable to metabolic studies [31]. In particular, CHO cells exhibited undistinguishable surface labelling upon either direct insertion of BODIPY-SM at 4°C, or as an exclusive bioconversion product from BODIPY-ceramide at the Golgi complex, providing a major argument against artificial self-assembly or aggregation. We therefore recently raised the hypothesis that BODIPY-SM micrometric domains depend on, and might reflect some preexisting compartmentation of, endogenous SM. Depletion of endogenous SM by SMase indeed prevented BODIPY-SM micrometric domains formation (Fig. 8) and decreased its mobile fraction in large fields to the level now observed for BODIPY-PC ([31], see Fig. 11D). In contrast, we now found that SM depletion (by SMase alone) left cells still competent to form BODIPY-PC micrometric domains (Fig. 8c), unless combined with GSL depletion by FB1 (Fig. 8d).

Moreover, we observed in this and our previous report [31] very similar micrometric assemblies by confocal imaging and undistinguishable FRAP properties, irrespectively of labelling a given lipid core by two different BODIPY spectral variants or by NBD. Furthermore, BODIPY-SM and GSLs are based on the very same BODIPY-ceramide backbone, yet showed clearcut differences which could only be explained by their distinct polar headgroups. Finally, we here reported that comparable micrometric domains were observed with a GPI-anchored red fluorescent protein reporter, for which the lipid moiety is fully intact and the fluorophore exlusively stands out of the bilayer, and that these showed differential co-localization with BODIPY-SM or -GlcCer, depending of the temperature.

### Coexistence of three distinct types of micrometric BODIPY-lipid assemblies: spatial segregation and additivity

Two lines of evidence support the coexistence of three distinct compartments made of micrometric assemblies, respectively enriched (up to 5- or even -8 fold; [31]) in fluorescent analogs of (i) three natural (D-*e*-) GSLs, GlcCer, LacCer and GM1; (ii) SM; and (iii) PC (or the artificial L-*t*-LacCer stereoisomer). Firstly, combinations of double-labelling experiments on CHO cells, thanks to the lipid analogs bearing the red spectral BODIPY we had synthesized, showed excellent co-localization of BODIPY^505^-LacCer with BODIPY^589^-GlcCer, but almost complete segregation from BODIPY^589^-SM ([31], Fig. 8). Likewise, in erythrocytes, while BODIPY^505^- and BODIPY^589^-GlcCer and the two GSLs, BODIPY^505^-GM1 and BODIPY^589^-GlcCer, extensively co-localized, BODIPY^589^-GlcCer and BODIPY^505^-PC were mostly segregated. Taken together, these straightforward observations already implied that three types of assemblies coexist. This conclusion is further supported by the differential localization of BODIPY-PC, -SM and -GlcCer micrometric assemblies with respect to the GPI-anchored red fluorescent protein reporter (Fig. 7). Yet, these three compartments might not be completely independent of one another, since BODIPY^589^-SM domains were preferably seen next to BODIPY^505^-LacCer domains ([31], Fig. 8C). Convoluted brilliant “round waves” of L_d_-tracers around non-enriched areas (see indentations indicated by red arrowheads at Figs. 4,5 and Fig. S3) raise the possibility of passive assembling due to preferential repulsion from L_o_-domains, but this hypothesis must be regarded as speculative at this time. Due to the particularly regular round contours of patches on erythrocytes, there was no indentation of lipid analogs domains, possibly due to a much higher cholesterol content or membrane tension (Fig. 1).

Secondly, whereas single labelling of CHO cells at 20°C with BODIPY^505^-PC, -SM or -GlcCer each labelled ∼25% of the cell surface, simultaneous labelling resulted into additive coverage, up to 70%. Lower coverage was observed on erythrocytes. Since temperature has a differential effect on BODIPY-lipid micrometric assemblies, 20°C was selected as a compromise: (i) for which all three types of domains could be readily observed on CHO cells; (ii) that could be rapidly reached by warming up; and (iii) at which imaging could be rapidly performed before significant internalization would take place. This type of experiment could not be performed in untreated cells at 37°C. On the other hand, full inhibition of endocytosis, by either energy depletion or K^+^-depletion [31], could interfere with domains structure. Thus, the respective contribution of the three compartments to the CHO cell surface at 37°C remains unknown, but the temperature-dependence for the three derivatives on the endocytosis-defective erythrocytes suggests that contribution of GSLs-enriched domains could become predominant at 37°C.

### Distinct thermodynamic properties of the different compartments: differential L_d_- *vs* L_o_-partitioning and restriction to lateral mobility between PC- and GSLs-analogs

Two thermodynamic properties independently allowed to distinguish the lipid analogs studied: phase partitioning and lateral mobility. The various lipid analogs of PC and GSLs used here showed clearly distinct partitioning related to their lipid moiety. Firstly, confocal analysis in erythrocytes revealed distinct temperature-dependence for BODIPY-PC (low temperature) *vs* -GlcCer (physiological temperature) assemblies, as predicted from respective Tm values for natural PC and GSL [35], [36]. Secondly, as is also known for their natural counterparts, BODIPY-PC, -SM and -GlcCer showed differential clustering in assemblies of increasing order, evidenced by the same differential propensity for excimer formation in erythrocytes and CHO cells. Thirdly, double-labelling in erythrocytes revealed an extensive co-localization between fluorescent -GlcCer and -GM1 but a large dissociation between -GlcCer and -PC. Fourthly, BODIPY-PC, -SM and -D-*e*-LacCer show differential localization with the unrelated L_o_-probe, the GPI-mRFP, further indicating their clustering into domains of differential ordering: no association for BODIPY-PC *vs* selective association with BODIPY-SM at 20°C and -D-*e*-LacCer at 37°C. These four independent arguments provide strong evidence that micrometric assemblies of PC-, SM- and GSL-analogs cannot be simply artefacts due to substitution of alkyl chains by fluorophores. In addition, lateral diffusion of PC- and L-*t*-LacCer-, but not of GSLs-, analogs was restricted in large fields at the PM of CHO cells at 30°C, but this restriction was largely relaxed at 37°C. This difference can be reasonably explained if the plasma membrane remains organized at physiological temperature in at least three lipid phases of differential ordering, with the continuous phase enriched in PC analogs. Such a model is in agreement with the lower restriction to its lateral mobility. Because of the lower order of the PC phase, SM and GSL analogs could cross-over the PC phase before reaching the bleached area.

We therefore raise the hypothesis that distinct fluorescent lipid partitioning could reflect differential intrinsic propensity of endogenous membrane lipids to form large assemblies. Obviously, since BODIPY-SM and -PC bearing the same phosphocholine headgroup can be spatially and functionally distinguished, the lipid interfacial region must play a major role. However, self-assembly must also be based, at least in significant part, on the *nature* or *orientation* of polar headgroups. Indeed, despite build-up on the same ceramide backbone, BODIPY-SM was largely segregated, both spatially and functionally, from BODIPY-GSLs (irrespectively if these were bearing mono- [GlcCer] or di- [D-*e*-LacCer] saccharides), the only difference left is thus in *nature* between zwitterionic *vs* neutral headgroups. The distinct morphological and dynamical properties of BODIPY-L-*t*-LacCer in comparison to D-*e*-LacCer also underline the importance of *orientation* of headgroups and backbones.

### A Speculative model of triple BODIPY-lipids PM compartmentation, based on endogenous L_o_-SM and “gel-like”-GSLs self-assemblies

We will finally develop a speculative model expanding in two directions on our recent proposal that fluorescent SM analogs could reveal preexisting domains stabilized by high SM concentrations, based on the capacity of BODIPY-SM domains to form excimers, indicating ordering, and on the dependence on the endogenous L_o_-SM for BODIPY-SM assembly and lateral diffusion properties [31]. Firstly, we further suggest that cohesion of SM analogs/endogenous SM assemblies would restrict access, thus mobility, for the L_d_-phase, the only left to accomodate the bulk of BODIPY-PC (and the artificial L-*t*-LacCer). In our view, this new proposal best explains why SM depletion by SMase not only causes disappearance of micrometric BODIPY-SM compartmentation and impairs its mobility (both being reversed by SM replenishment; [31]), but simultaneously leads to partial dispersion of micrometric assemblies of BODIPY-PC and -L-*t*-LacCer (Fig. 8c,c′ and Fig. S4), while relaxing the restriction to their lateral diffusion (Fig. 11 and data not shown).

Secondly, we further evidenced a phase separation between GSL and SM analogs, based on spatial segregation at 37°C [31] and differential colocalization with the L_o_-reporter GPI-mRFP depending on relatively small changes in temperature (30°C *vs* 37°C). Indeed, like natural SLs, where Tm values are higher for GSLs than for SM, BODIPY-GSLs formed micrometric domains at higher temperature (these were the most abundant domains on erythrocytes at 37°C, Fig. 1C; and the best defined on CHO cells, Fig. 5f,f′) and showed a higher propensity than BODIPY-SM to produce excimers. We thus favor the view that GSLs fluorescent analogs (and their endogenous conterparts) would form the most robust and selective micrometric domains, which should be poorly permeable to SM and even less to PC, but readily traverse as structured assemblies the less ordered SM phase and find no obstacle in the L_d_-phase. This proposal could explain why only BODIPY-GSLs showed no difference in lateral mobility between small and large photobleached fields. Conversely, less ordered SM domains would be restricted by GSLs domains but not by the L_d_-phase, thus show partial restriction, and the L_d_-tracers such as analogs of PC (and the artificial L-*t*-LacCer) would be maximally restricted in untreated cells. This restriction would be partially relaxed upon removal of SM (raising mobile fraction from 25% to 35%) and fully relaxed by combined SL depletion by fumonisin B1 (mobile fraction up to 60%).

Our observation that two SL analogs sharing the same ceramide backbone, BODIPY-SM and -GSLs, partition into distinct micrometric domains could seem surprising but is in full agreement with recent reports on the differential organization of SM and GSLs. In fluid model membranes, SM forms in presence of high cholesterol L_o_-domains with similar properties to rafts in biological membranes, whereas saturated GSLs can form “gel-phase” microdomains even without cholesterol. Moreover, in living cells, different SLs can segregate into different PM domains (for a review, see [9]).

We do not wish to speculate further on the general micrometric membrane compartmentation hypothesis, nor on its possible relation with polarity [47], [48], budding yeast [49], and vesicular fusion or fission [50]. As a priority, we currently test the relation of BODIPY-lipid micrometric domains with cholesterol levels (graded depletion *vs* graded overload). Whether a similar fine modulation of SM and GSLs by selective replenishment can also be achieved [31] deserves to be explored more precisely. Alternatively, CHO mutants with altered levels of SLs may be more thoroughly studied, although the extent of depletion could not be sufficient for reproducible differences to be recorded. Measures of actual Tm values of the lipid analogs by comparison with their natural counterparts are, of course, essential. Whether similar domains can be found on the yeast PM also deserves to be clarified.

## Materials and Methods

Most methods are described in details elsewhere [31].

### Commercial lipids, reagents and synthesis of fluorescent lipids

1-oleoyl-2-[6-[(7-nitro-2-1,3-benzoxadiazol-4-yl)amino]hexanoyl]-sn-glycero-3-phosphocholine (NBD-phosphatidyl-choline [C18:1]), glucopsychosine, lactosylsphingosine and lyso-sphingomyelin were from Avanti polar lipids. NBD-glucosylceramide, D-PDMP, L-PDMP, ceramides, sphingomyelins, glucocerebrosides, phosphatidylethanolamine were from Matreya. BODIPY-D-erythro N-(4,4-difluoro-5,7-dimethyl-4-bora-3a,4a-diaza-s-indacene-3-pentanoyl)sphingosyl1-β-D-lactoside (BODIPY-LacCer) and -L-threo-BODIPY-LacCer were a kind gift of Professor D. Pagano [3]. All other fluorescent lipids, Alexa 488-cholera toxin B subunit, N-hydroxysuccinimidoyl ester of 4,4-difluoro-5,7-dimethyl-4-bora-3a,4a-diaza-s-indacene-3-pentanoic acid (referred to as BODIPY-fatty acid) were from Invitrogen. Poly-L-lysine (70–150 kDa), *Bacillus cereus* sphingomyelinase (SMase), fumonisin B1 (FB1), defatted bovine serum albumin (DF-BSA) were from Sigma-Aldrich. Latrunculin B and silica gel 60 F254 HP-TLC plates were from Merck. SM, GlcCer and D-*e*-LacCer were conjugated to BODIPY by *N*-acylation [51] of lyso-sphingomyelin, glucosyl-β-sphingosine and lactosyl-β1-sphingosine respectively, using the *N*-hydroxysuccinimidyl ester of corresponding BODIPY-fatty acids, exactly as described [31].

### Preparation of human erythrocytes for live cell imaging

After written informed consent, two healthy donors regularly provided fresh erythrocytes. After blood collection by venopuncture into dry EDTA-coated tubes and dilution in Hank's Buffered Salt Solution (HBSS; pH 7.2), erythrocytes were isolated by centrifugation and resuspension as described [31], then allowed to adhere onto poly-L-lysine-coated coverslips at 20°C for 4–10 min. After three washes, coverslip-bound erythrocytes were incubated in HBSS at 20°C for another 5 min, then labelled with 0.5 to 3 µM BODIPY-lipids in HBSS containing DF-BSA (1∶1 lipid/BSA molar ratio) still at 20°C for 15 min. For NBD probes, attached erythrocytes were labelled with higher concentrations of NBD-PC in HBSS/DF-BSA at 20°C for 15 min (4 µM NBD-PC [16∶0], 8 µM NBD-PC [18∶1]). For double-labelling, erythrocytes were sequentially labelled with 4 µM BODIPY^589^-GlcCer at 37°C, then allowed to adhere to poly-L-lysine before labeling with BODIPY^505^-lipids in the continued presence of BODIPY^589^-GlcCer at 20°C. After 5 rapid washes, all coverslips were then placed bottom-up into Lab-Tek chambers so as to allow direct viewing by the objective of the free upper cell surface at the designed temperature (10°C, 20°C, 30°C or 37°C; for control of temperature, see below). Alternatively, the entire procedure for erythrocytes labelling, washing and imaging with BODIPY-lipids was conducted at 37°C.

### CHO cells culture, transfection and pharmacological treatments

CHO cells were propagated in DMEM/F12 medium with 10% FCS as described [31]. For experiments, cells were seeded at 20,000/cm^2^ and grown till ∼90% confluency (2days) on Lab-Tek chambers (Nunc, Roskilde, Denmark) for live cell imaging and FRAP, or Petri dishes for biochemical experiments. CHO cells were transfected with GPI-mRFP plasmid [39] using lipofectamine exactly as described [31]. When appropriate, before surface labelling, CHO cells were preincubated at 37°C in medium with 50 nM latrunculin B for 30 min; with 0.1 U/ml SMase for 30 min; with 20 µM D-PDMP (or L-PDMP as negative control) for 24 h; or with 30 µM FB1 for 48 h (the two latter in medium with serum). For efficiency of depletion, see Table S1. None of these treatments caused detectable toxicity, nor significantly altered PM incorporation of lipid analogs. To block endocytosis, CHO cells were K^+^-depleted [52], as validated in [31]. Live cell imaging and FRAP analysis were performed in the continued presence of latrunculin B or K^+^-depleting medium, as appropriate.

### Live cell imaging and FRAP

Dried lipid analogs were dissolved in absolute ethanol and mixed with culture medium or HBSS enriched with equimolar DF-BSA and 25 mM HEPES, pH 7.1, under vigorous vortexing, then cleared of possible aggregates by centrifugation at 14,000×*g* for 5 min. The following lipid analogs were used in DF-BSA (1∶1), with final ethanol <0.5%: 0.5 or 1 µM BODIPY-lipids (except for excimer studies), 5 µM NBD-GlcCer, 10 µM NBD-PC. For cell labelling, cells were usually placed into ice-cold culture medium for 10 min then incubated with lipid analogs at 4°C for 30 min. After surface labelling, cells were washed 5 times with serum-free medium at 4°C, then analyzed by confocal microscopy or FRAP immediately after transfer to the desired temperature. When specified, cells were directly incubated at 20°C, 30°C or 37°C for 15 min and washed at the same temperature before analysis. All morphological studies were performed by vital imaging using Plan-Apochromat 63x/1.4 oil immersion objectives. After surface-labeling with fluorescent lipids (at 4°C, except otherwise stated), cells were washed, briefly transferred to medium adjusted to 10°C, 20°C, 30°C or 37°C as indicated, and immediately imaged by confocal microscopy exactly at the same temperature. Image acquisition and FRAP were performed using either a Bio-Rad MRC1024/Zeiss Axiovert M135 confocal microscope equipped with a cooling/warming stage adjusted to reach 10°C in the observation chamber (Lauda Ecoline Staredition RE106; Lauda-Königshofen, Germany), or a Zeiss LSM510 confocal microscope set at 20°C, 30°C or 37°C (XL/LSM incubator, Zeiss). Imaging and FRAP patterns were essentially superimposable with the two settings, although mobile fraction values were consistently higher with the Zeiss LSM510 equipment. For erythrocytes, the focal plane was always set at the center of the free surface, at which the number of brilliant patches was counted (also referred to as “hemi-erythrocyte surface”). For CHO cells, imaging was recorded at the bottom (flat) PM, to minimize surface anatomical features and to avoid saturation of the lateral PM signal [31]. Intensity profiles were recorded along the most informative paths, indicated in orange and well-defined patches (or non-labelled zones) were numbered on confocal images to identify corresponding peaks or nadirs in the line intensity profiles. For both BODIPY- and NBD-lipids, images were recorded at 0.3-2% laser power. For double labelling, data were sequentially acquired in the green (λexc 488 nm) then in the red channel (λexc 568 nm; at 8-10% laser power). For excimer studies, erythrocytes were excited at 488 nm and images were simultaneously acquired in the green (λem 520 nm) and red channels (λem 605 nm); CHO cells were excited at 488 nm (λexc 488 nm) and 568 nm (λexc 568 nm) and images were acquired in the green and red channels then merged. Fluorescence recovery after photobleaching (FRAP) was performed exactly as described [31].

### Statistical analyses

All values are presented as means±SEM. FRAP values were adjusted to monoexponential fitting. Statistical significance of comparisons was tested by the Student's t test, using GraphPad Instat programs (GraphPad Software Inc., San Diego). NS, not significant; *, p<0.05; **, p<0.01; ***, p<0.001.

## Supporting Information

Figure S1 **Structure of lipid analogs used in the study.** **(A) Sphingolipid analogs.** The basic structures of the D-*e* (natural stereochemistry) or L-*t* (artificial stereochemistry) are shown above; polar heads are represented as R′: glucosyl, phosphocholine or lactosyl; fluorescent fatty acids are represented as R: BODIPY (boron dipyrromethenedifluoride), referring to the BODIPY^505^, unless stated otherwise, BODIPY^589^ (notice the more bulky fluorophore), or NBD (7-nitrobenz-2-oxa-1,3-diazol-4-yl). Glucosylceramide (GlcCer) was substituted at position #C_5_ of the fatty acid by BODIPY^505^ (BODIPY^505^-GlcCer), at #C_6_ by BODIPY^589^ (BODIPY^589^-GlcCer) and at #C_6_ by NBD (NBD-GlcCer). Sphingomyelin (SM) was also substituted at #C_5_ by BODIPY^505^ (BODIPY^505^-SM), at #C_6_ by BODIPY^589^ (BODIPY^589^-SM) and at #C_6_ by NBD (NBD-SM). Lactosylceramide with a natural stereochemistry (D-*e*) or artificial stereochemistry (L-*t*; the differential stereochemistry of hydroxyl group at C_3_ of sphingosine is emphasized in red) were both substituted at #C_5_ by BODIPY^505^ (BODIPY-D-*e*-LacCer and -L-*t*-LacCer). **(B) Glycerophospholipid analogs.** Saturated phosphatidylcholine (C16:0) was substituted at #C_5_ by BODIPY (BODIPY-PC [C16:0]) and at #C_6_ by NBD (NBD-PC [C16:0]); mono-unsaturated phosphatidylcholine (C18:1) was substituted at #C_6_ by NBD (NBD-PC [C18:1]). This figure is adapted from Fig. S1 of [31], for reader′s convenience.
(TIF)

Figure S2 **Micrometric BODIPY-GlcCer domains on erythrocytes are not a metastable state due to exposure at low temperature. (a)** Labeling of erythrocytes with BODIPY-GlcCer and washing at room temperature, followed by imaging at 37°C (reproduced from [31]). **(b)** Entire procedure for erythrocyte labeling, washing and imaging of BODIPY-GlcCer at 37°C. Scale bars, 2** µ**m.
(TIF)

Figure S3 **BODIPY-PC and -GSL peripheral patches do not reflect endocytosis nor depend on cortical actin.** CHO cells were either kept untreated (CTL; a,d), K^+^-depleted (-K^+^; b,e) or treated with latrunculin B (LatB; c,f). Thereafter, cells were surface-labelled with BODIPY-PC or with BODIPY-D-*e*-LacCer, washed and bottom confocal sections were recorded at ∼20 min after transfer to 37°C. All scale bars, 2** µ**m. The patchy surface distribution of both lipid analogs remains after K^+^-depletion and latrunculin B treatment. Notice convoluted labelling for both lipid analogs, with notches indicated by red arrowheads.
(TIF)

Figure S4 **BODIPY-L-** ***t*** **-LacCer patches mimic BODIPY-PC for resistance to endogenous GSLs or SM depletion.** CHO cells were kept untreated (a, CTL), or treated with D-PDMP (b), SMase (c) or FB1 (d), then surface-labelled with BODIPY-L-*t*-LacCer, washed and bottom cell surface was directly imaged by confocal microscopy at 10°C using the same laser power. Scale bar, 2** µ**m. For comparison with BODIPY-PC, see Fig. 8, left.
(TIF)

Figure S5 **Co-localization of GPI-mRFP with BODIPY-SM at 20**°**C** ***vs*** **-D-** ***e*** **-LacCer at 37**°**C.** Extended presentation of Fig. 7, panels c-f, with additional data at 30°C. Single channel recordings allow to better evidence that co-localization with GPI-mRFP is restricted to a different temperature for the two SL analogs. Panels at 30°C reveal marked differences from 37°C.
(TIF)

Table S1 **Effect of treatments on endogenous lipids (residual lipids as % of untreated cells).** ^a,b^ To assay for levels of GlcCer, GM3, SM and ceramide (as reference), cells were metabolically labeled with 0.5** µ**Ci/ml 3H-palmitic acid for 3days, then total cell lipids were extracted [53] and resolved by TLC. Spots were excised and radioactivity was determined by liquid-scintillation counting and normalized: ^a^ GlcCer and GM3 contents are expressed by reference to the corresponding major band (phosphatidylethanolamine); ^b^ SM contents are normalized to ceramide. -, not tested. Values are averages of two or means**±**SEM when applicable (*from 2 to 4 experiments*).
(TIF)

Table S2 **Comparison of mobile fraction in small (5 µm^2^) and large (20 µm^2^) membrane fields.** The indicated fluorescent lipid probes were inserted into the plasma membrane of CHO cells. After washing at 4°C, small (5** µ**m^2^) or large (20** µ**m^2^) fields were photobleached and fluorescence recovery was measured at 30°C. Experimental values were fitted to monoexponentials, to derive mobile fractions at infinite time of recovery (Mf). Values are means±SEM (*number of experiments in parentheses*). ^a^ The statistical significance of differences was tested by reference to 5** µ**m^2^-fields (NS, not significant; ***, p<0.001). ^b^ Values were reproduced or are extended from Tyteca et al [31], for comparison purpose.
(TIF)
