# Supplementary figures and images for: Segregation of Fluorescent Membrane Lipids into Distinct Micrometric Domains: Evidence for Phase Compartmentation of Natural Lipids?
Source: PLoS One. 2011 Feb 28;6(2):e17021. doi: 10.1371/journal.pone.0017021 (PMC3046177; doi:10.1371/journal.pone.0017021)

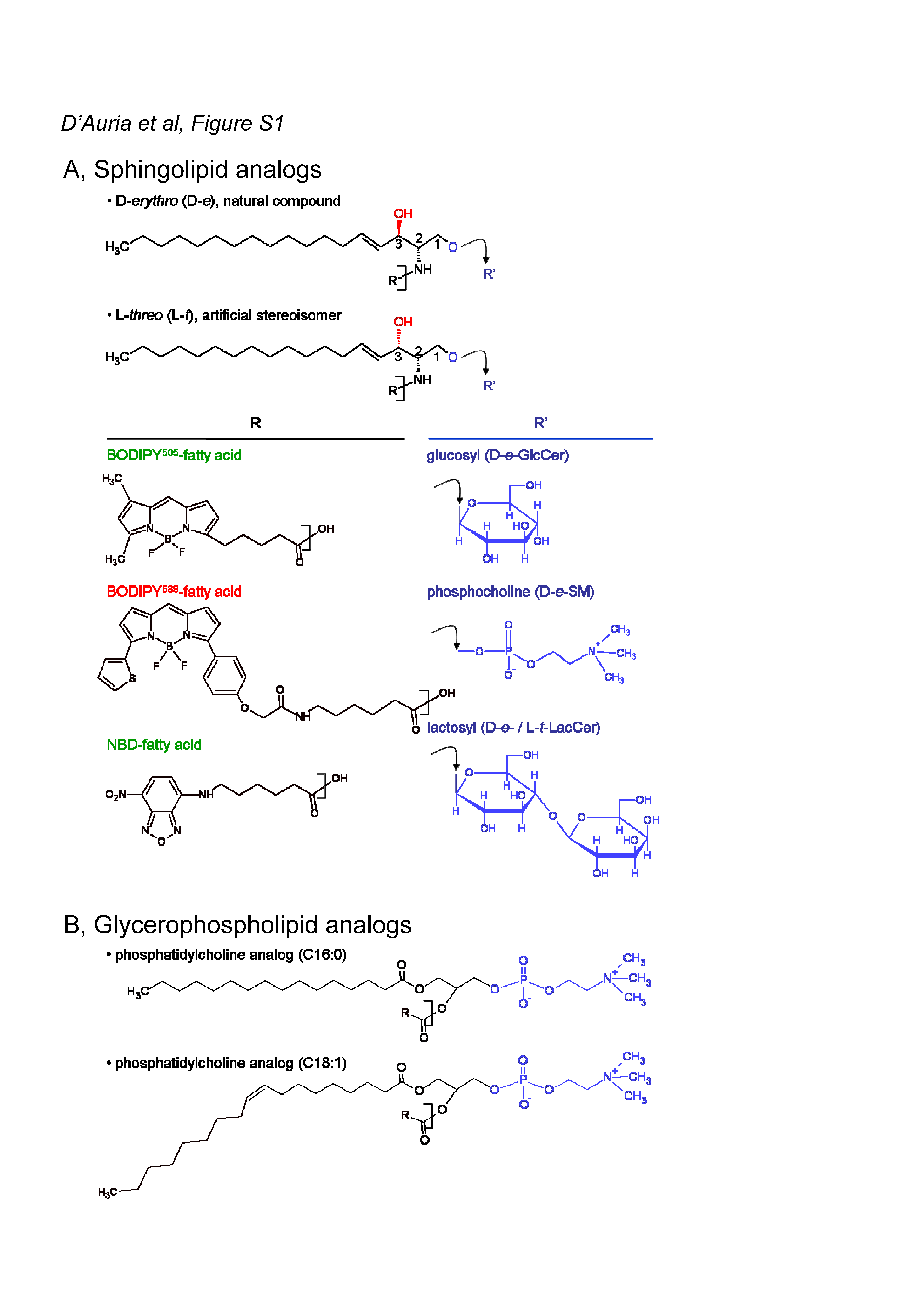

Supplement: Figure S1 — Structure of lipid analogs used in the study. (A) Sphingolipid analogs. The basic structures of the D-e (natural stereochemistry) or L-t (artificial stereochemistry) are shown above; polar heads are represented as R′: glucosyl, phosphocholine or lactosyl; fluorescent fatty acids are represented as R: BODIPY (boron dipyrromethenedifluoride), referring to the BODIPY505, unless stated otherwise, BODIPY589 (notice the more bulky fluorophore), or NBD (7-nitrobenz-2-oxa-1,3-diazol-4-yl). Glucosylceramide (GlcCer) was substituted at position #C5 of the fatty acid by BODIPY505 (BODIPY505-GlcCer), at #C6 by BODIPY589 (BODIPY589-GlcCer) and at #C6 by NBD (NBD-GlcCer). Sphingomyelin (SM) was also substituted at #C5 by BODIPY505 (BODIPY505-SM), at #C6 by BODIPY589 (BODIPY589-SM) and at #C6 by NBD (NBD-SM). Lactosylceramide with a natural stereochemistry (D-e) or artificial stereochemistry (L-t; the differential stereochemistry of hydroxyl group at C3 of sphingosine is emphasized in red) were both substituted at #C5 by BODIPY505 (BODIPY-D-e-LacCer and -L-t-LacCer). (B) Glycerophospholipid analogs. Saturated phosphatidylcholine (C16:0) was substituted at #C5 by BODIPY (BODIPY-PC [C16:0]) and at #C6 by NBD (NBD-PC [C16:0]); mono-unsaturated phosphatidylcholine (C18:1) was substituted at #C6 by NBD (NBD-PC [C18:1]). This figure is adapted from Fig. S1 of [31], for reader′s convenience. (TIF) [file pone.0017021.s001.tif]

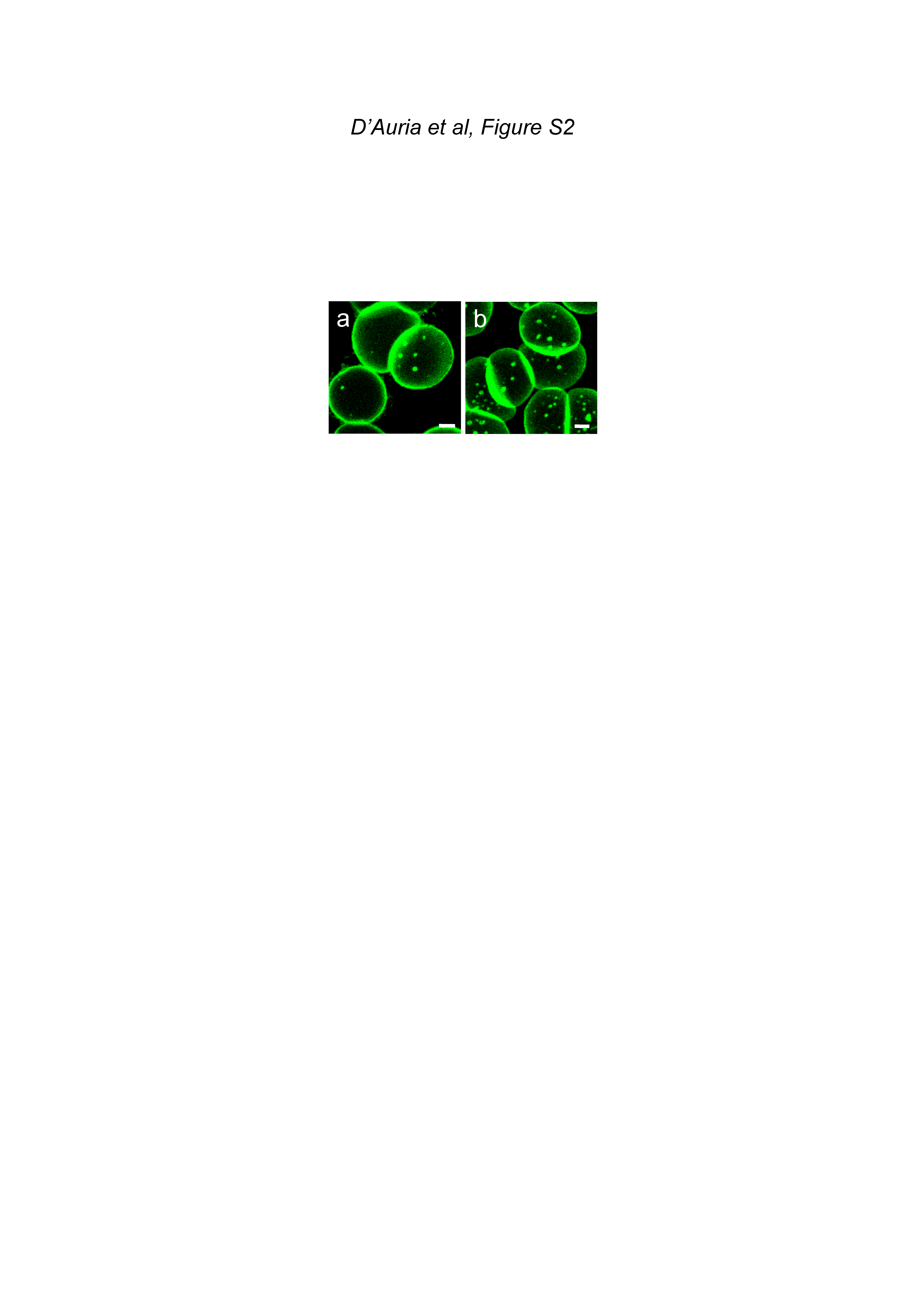

Supplement: Figure S2 — Micrometric BODIPY-GlcCer domains on erythrocytes are not a metastable state due to exposure at low temperature. (a) Labeling of erythrocytes with BODIPY-GlcCer and washing at room temperature, followed by imaging at 37°C (reproduced from [31]). (b) Entire procedure for erythrocyte labeling, washing and imaging of BODIPY-GlcCer at 37°C. Scale bars, 2 µm. (TIF) [file pone.0017021.s002.tif]

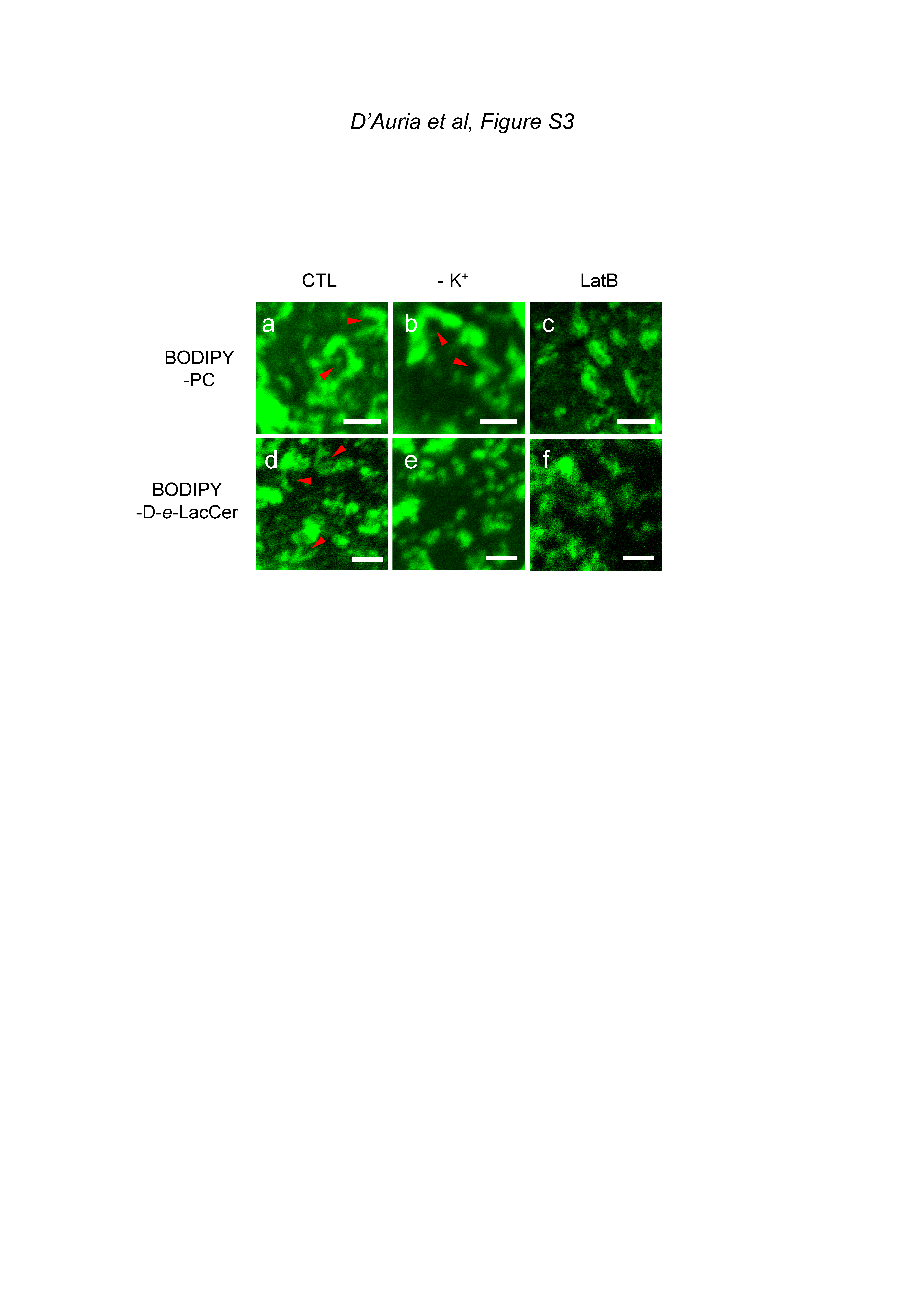

Supplement: Figure S3 — BODIPY-PC and -GSL peripheral patches do not reflect endocytosis nor depend on cortical actin. CHO cells were either kept untreated (CTL; a,d), K+-depleted (-K+; b,e) or treated with latrunculin B (LatB; c,f). Thereafter, cells were surface-labelled with BODIPY-PC or with BODIPY-D-e-LacCer, washed and bottom confocal sections were recorded at ∼20 min after transfer to 37°C. All scale bars, 2 µm. The patchy surface distribution of both lipid analogs remains after K+-depletion and latrunculin B treatment. Notice convoluted labelling for both lipid analogs, with notches indicated by red arrowheads. (TIF) [file pone.0017021.s003.tif]

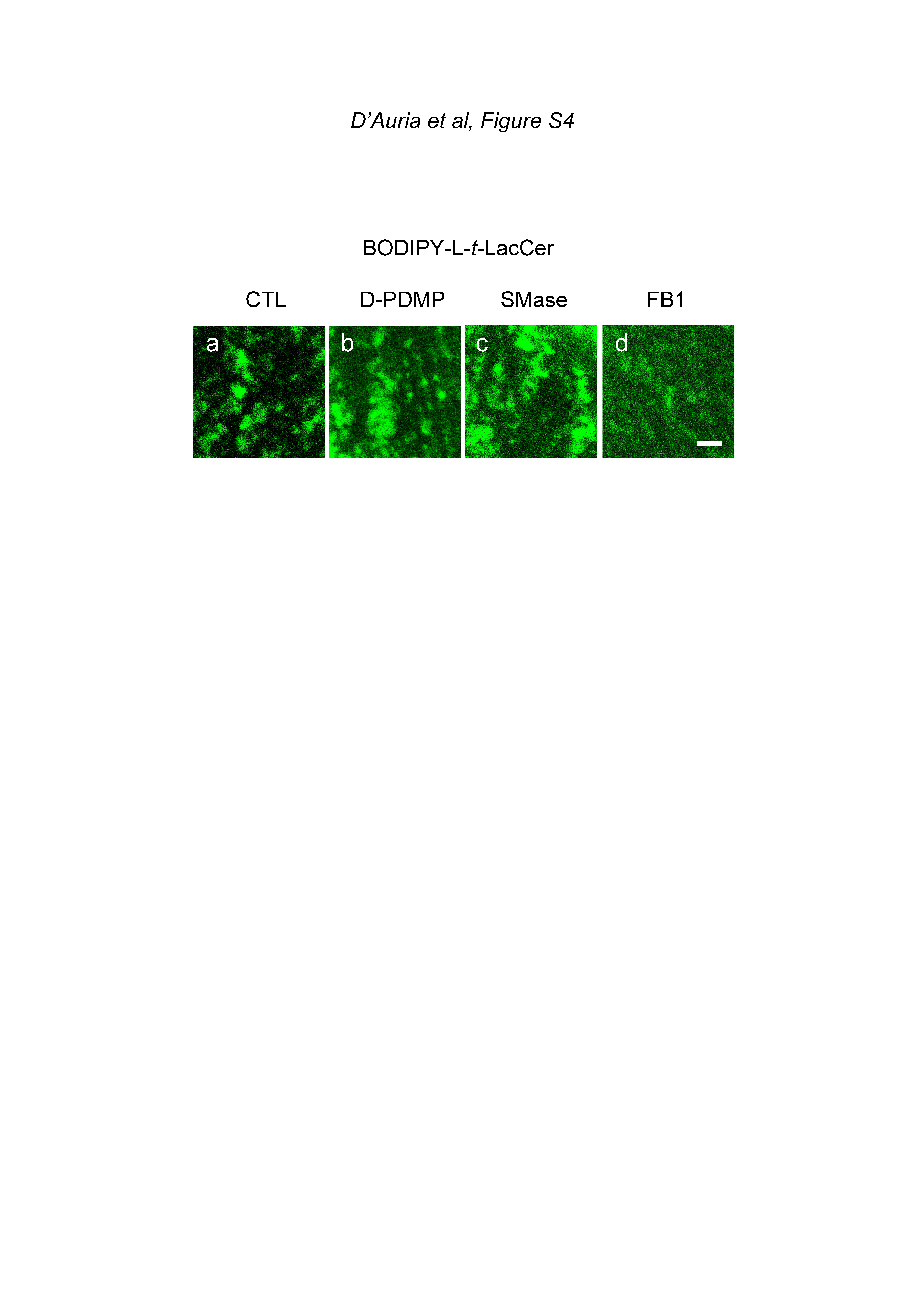

Supplement: Figure S4 — BODIPY-L- t -LacCer patches mimic BODIPY-PC for resistance to endogenous GSLs or SM depletion. CHO cells were kept untreated (a, CTL), or treated with D-PDMP (b), SMase (c) or FB1 (d), then surface-labelled with BODIPY-L-t-LacCer, washed and bottom cell surface was directly imaged by confocal microscopy at 10°C using the same laser power. Scale bar, 2 µm. For comparison with BODIPY-PC, see Fig. 8, left. (TIF) [file pone.0017021.s004.tif]

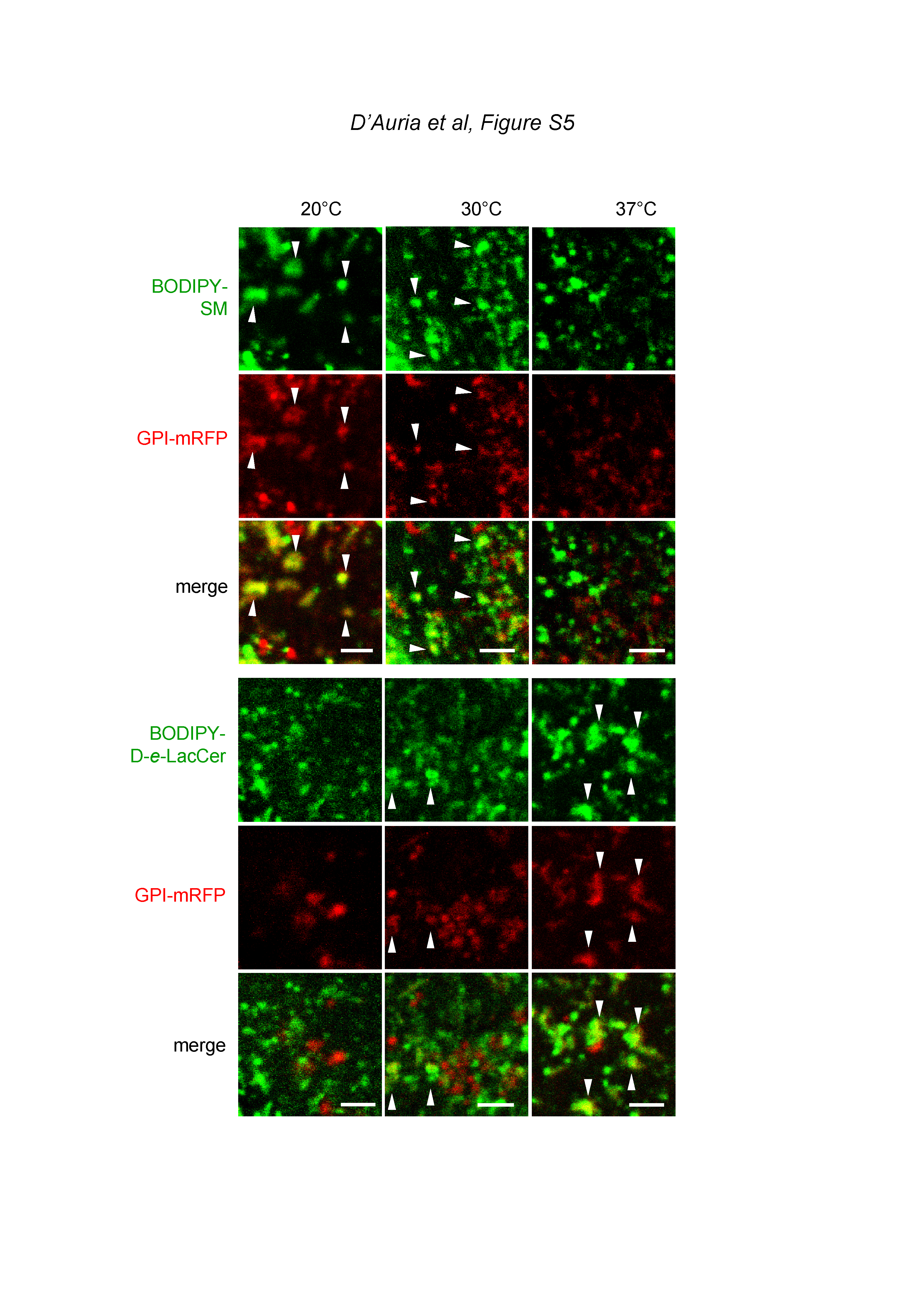

Supplement: Figure S5 — Co-localization of GPI-mRFP with BODIPY-SM at 20°C vs -D- e -LacCer at 37°C. Extended presentation of Fig. 7, panels c-f, with additional data at 30°C. Single channel recordings allow to better evidence that co-localization with GPI-mRFP is restricted to a different temperature for the two SL analogs. Panels at 30°C reveal marked differences from 37°C. (TIF) [file pone.0017021.s005.tif]

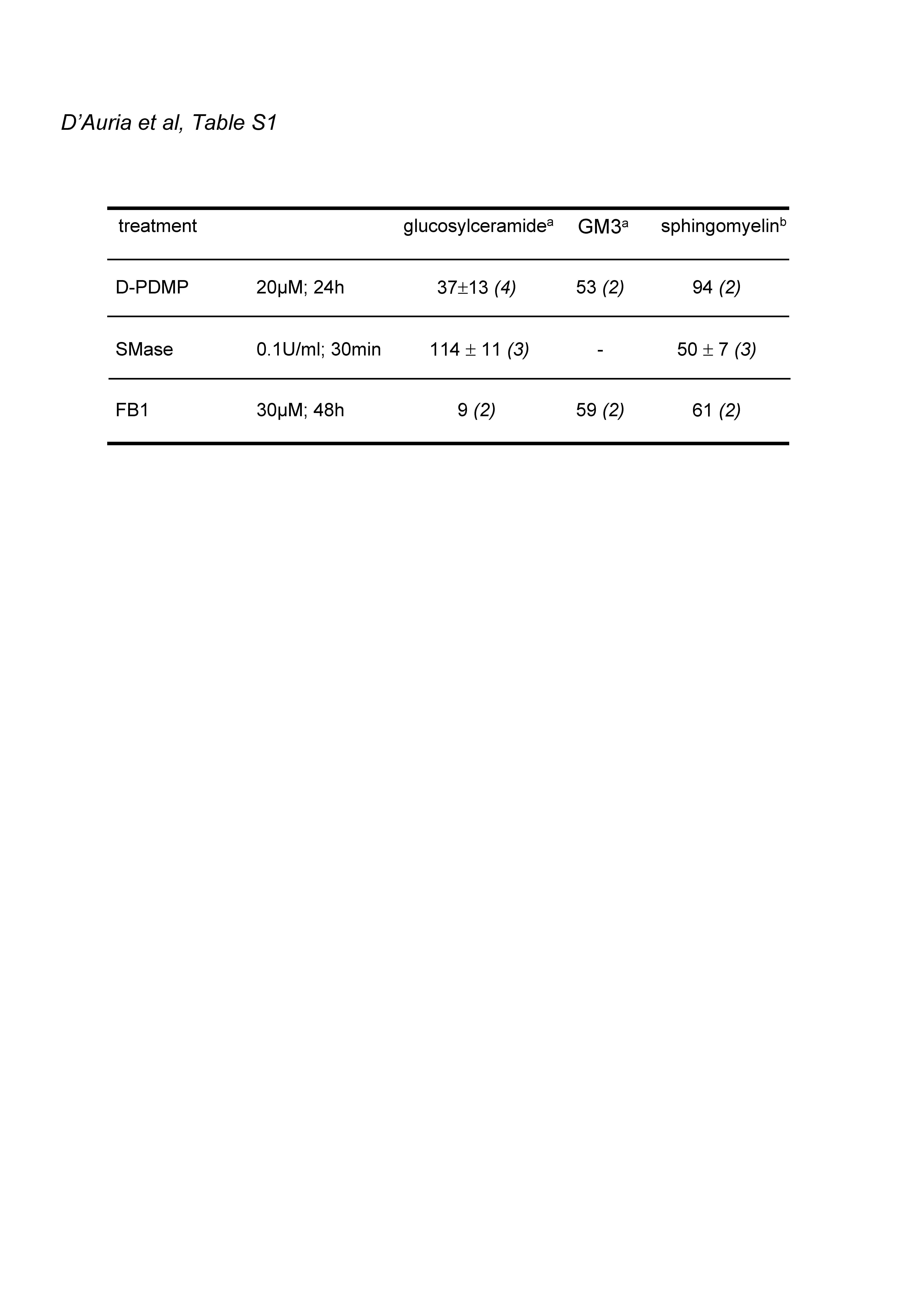

Supplement: Table S1 — Effect of treatments on endogenous lipids (residual lipids as % of untreated cells). a,b To assay for levels of GlcCer, GM3, SM and ceramide (as reference), cells were metabolically labeled with 0.5 µCi/ml 3H-palmitic acid for 3days, then total cell lipids were extracted [53] and resolved by TLC. Spots were excised and radioactivity was determined by liquid-scintillation counting and normalized: a GlcCer and GM3 contents are expressed by reference to the corresponding major band (phosphatidylethanolamine); b SM contents are normalized to ceramide. -, not tested. Values are averages of two or means±SEM when applicable (from 2 to 4 experiments). (TIF) [file pone.0017021.s006.tif]

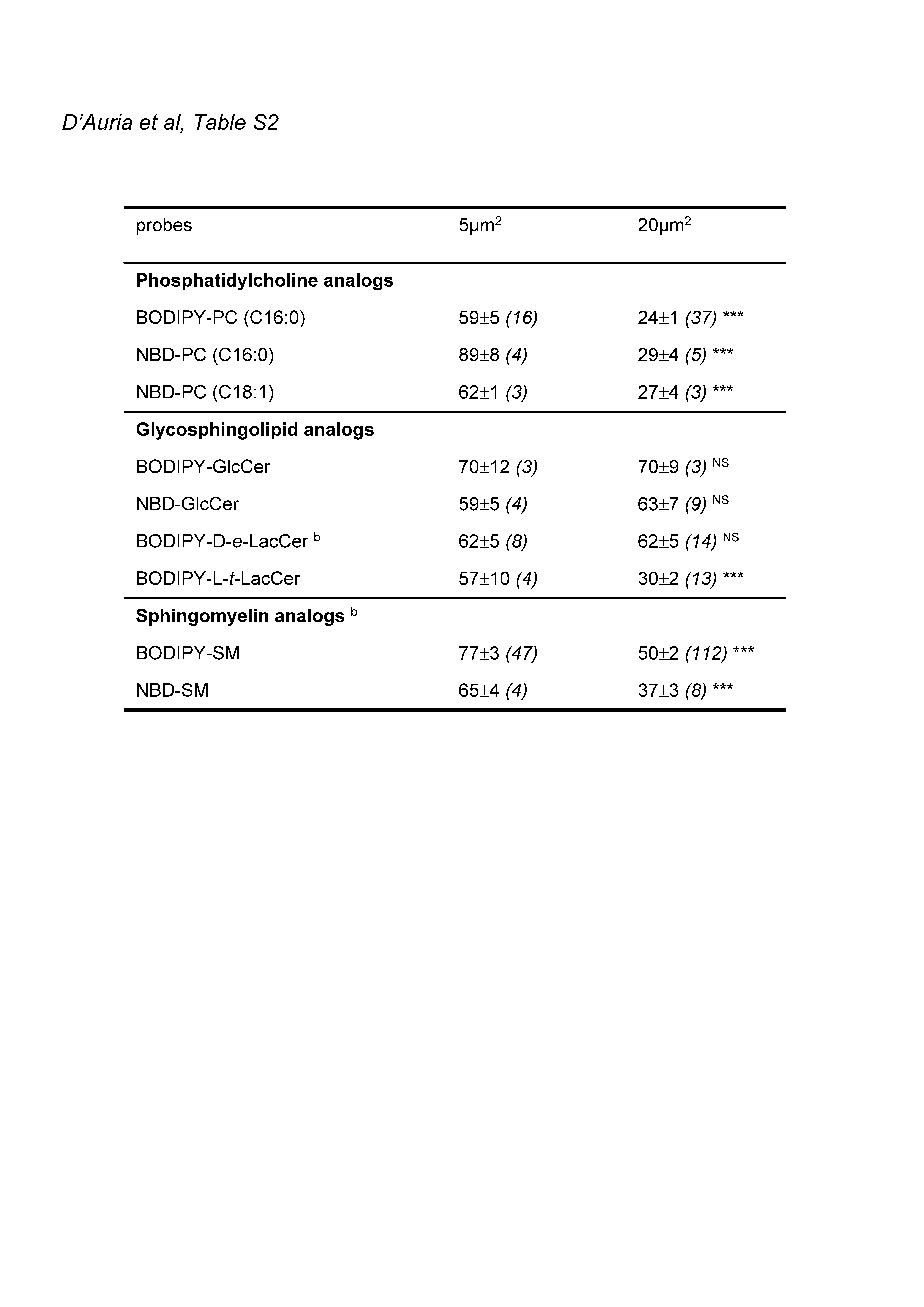

Supplement: Table S2 — Comparison of mobile fraction in small (5 µm2) and large (20 µm2) membrane fields. The indicated fluorescent lipid probes were inserted into the plasma membrane of CHO cells. After washing at 4°C, small (5 µm2) or large (20 µm2) fields were photobleached and fluorescence recovery was measured at 30°C. Experimental values were fitted to monoexponentials, to derive mobile fractions at infinite time of recovery (Mf). Values are means±SEM (number of experiments in parentheses). a The statistical significance of differences was tested by reference to 5 µm2-fields (NS, not significant; ***, p<0.001). b Values were reproduced or are extended from Tyteca et al [31], for comparison purpose. (TIF) [file pone.0017021.s007.tif]
